# Two-Dimensional Perisaccadic Visual Mislocalization in Rhesus Macaque Monkeys

**DOI:** 10.1523/ENEURO.0547-24.2025

**Published:** 2025-06-04

**Authors:** Matthias P. Baumann, Ziad M. Hafed

**Affiliations:** ^1^Werner Reichardt Centre for Integrative Neuroscience, University of Tübingen, Tübingen 72076, Baden-Württemberg, Germany; ^2^Hertie Institute for Clinical Brain Research, University of Tübingen, Tübingen 72076, Baden-Württemberg, Germany

**Keywords:** active vision, perception, perisaccadic compression, perisaccadic mislocalization, saccades, visual stability

## Abstract

Perceptual localization of brief, high-contrast perisaccadic visual probes is grossly erroneous. While this phenomenon has been extensively studied in humans, more needs to be learned about its underlying neural mechanisms. This ideally requires running similar behavioral paradigms in animals. However, during neurophysiology, neurons encountered in the relevant sensory and sensory–motor brain areas for visual mislocalization can have arbitrary, noncardinal response field locations. This necessitates using mislocalization paradigms that can work with any saccade direction. Here, we first established such a paradigm in three male rhesus macaque monkeys. In every trial, the monkeys generated a saccade toward an eccentric target. Once a saccade onset was detected, we presented a brief flash at one of three possible locations ahead of the saccade target location. After an experimentally imposed delay, we removed the saccade target, and the monkeys were then required to generate a memory-guided saccade toward the remembered flash location. All three monkeys readily learned the task, and, like humans, they all showed strong backward mislocalization toward the saccade target, which recovered for later flashes from the saccade time. Importantly, we then replicated a known property of human perisaccadic mislocalization, as revealed by two-dimensional mislocalization paradigms: that mislocalization is the strongest for upward saccades. For horizontal saccades, we additionally found stronger mislocalization for upper visual field flashes, again consistent with humans. Our results establish a robust two-dimensional mislocalization paradigm in monkeys, and they pave the way for exploring the neural mechanisms underlying the dependence of perisaccadic mislocalization strength on saccade direction.

## Graphical abstract

**Figure:**
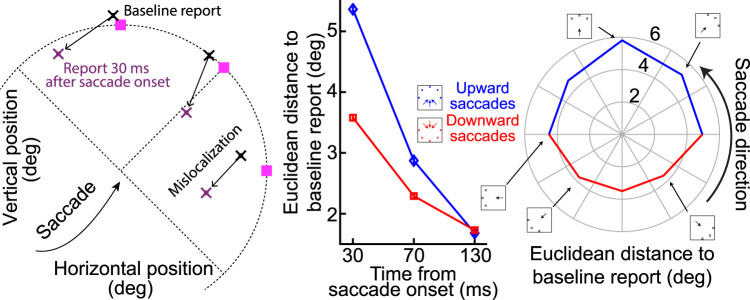
Visual Abstract

## Significance Statement

Visual perception is strongly altered around the time of saccades. Such alteration is often studied by characterizing how brief perisaccadic visual flashes are perceptually localized. While the properties of visual mislocalization have been exhaustively studied, the underlying neural mechanisms remain elusive, and this is due to a lack of suitable behavioral paradigms in animal models. We describe such a paradigm for macaques, which are ideal for exploring sensory–motor neural processes related to perisaccadic mislocalization.

## Introduction

Studying visual perception requires investigating the interplays between visual sensory processing and eye movements. This is because eye movements, such as saccades, can drastically alter retinal images even if the external environment is completely stable.

Multiple alterations in visual information processing are known to accompany saccades. For example, perisaccadic visual sensitivity is reduced, sometimes rendering brief visual presentations completely invisible (Latour, 1962; Zuber and Stark, 1966; Beeler, 1967). Moreover, when stimulus contrasts are high enough to be detected, these stimuli are grossly mislocalized (Honda, 1989; Cai et al., 1997; Ross et al., 1997). Such mislocalization depends on various factors related to the visual environment (Lappe et al., 2000). For example, in complete darkness, brief presaccadic flashes are perceived to have appeared ahead of their true positions (Dassonville et al., 1992; Lappe et al., 2000); such mislocalization is often referred to as forward mislocalization, since the error in reported position has a vector of similar direction to the saccade vector direction (Klingenhoefer and Krekelberg, 2017). Interestingly, the mislocalization is biphasic (Honda, 1991, 1993), with postsaccadic flashes being perceived as having been shifted opposite, rather than along, the saccade vector. On the other hand, with visual references, perisaccadic mislocalization is no longer biphasic but dominated by a “compression” toward the saccade target: brief flashes presented ahead of the saccade target experience mislocalization in the opposite direction from the saccade, even presaccadically (Ross et al., 1997; Klingenhoefer and Krekelberg, 2017); flashes presented in between the initial gaze position and the saccade target undergo forward mislocalization. Such “compression” could reflect the importance of the saccade goal in updating visual–motor reference frames (Schoppik and Lisberger, 2006; Zirnsak et al., 2014), and it implies that in structured visual environments, observing forward or backward mislocalization is no longer a function of time (like in complete darkness) but a function of spatial location (Honda, 1999).

Even though much of the early work on perisaccadic mislocalization has focused on only one dimension (horizontal saccades and perceptual reports on only the horizontal position of flashes), subsequent pioneering work demonstrated that, even with horizontal saccades, mislocalization has a distinct two-dimensional landscape (Kaiser and Lappe, 2004). More importantly, saccade directions themselves alter the two-dimensional mislocalization landscape, with upward saccades causing particularly strong reverse mislocalization for flashes presented ahead of the saccade target (Grujic et al., 2018). This latter result, which was motivated by a difference between upper and lower visual field representations in the superior colliculus (Hafed and Chen, 2016; Zhang et al., 2022; Hafed, 2025), suggests a clear need to study perisaccadic mislocalization, whether perceptually or with neurophysiological experiments, using more than just horizontal saccades.

Here, we aimed to establish a robust and flexible perisaccadic visual mislocalization paradigm in macaque monkeys, setting the stage for subsequent neurophysiological experiments. Four previous studies demonstrated perisaccadic mislocalization in monkeys, but all used only horizontal saccades and with only one-dimensional mislocalization measures (analyzing the horizontal component of flash positions). In one study, forward mislocalization was observed in complete darkness (Dassonville et al., 1992), consistent with human studies (Honda, 1989; Lappe et al., 2000). In another, backward mislocalization was observed in the absence of complete darkness (even for flashes behind the saccade target location), inconsistent with so-called compression (Jeffries et al., 2007). However, the flashes used in that study were not the very brief flashes typical of mislocalization experiments in humans. In the third study, perisaccadic “compression” was observed like in humans and under identical experimental conditions between species (Klingenhoefer and Krekelberg, 2017), and, most recently, a spatial bias by the saccade target was documented, enabling simultaneous recordings of visual cortical neurons during the behavior (Weng et al., 2024). Thus, it is indeed plausible to use monkeys for mislocalization experiments.

Having said that, the reality of neurophysiological experiments is that saccade direction could be completely arbitrary. For example, if one were to record superior colliculus saccade motor bursts, as a potential source of corollary discharge signals (Sommer and Wurtz, 2004, 2006; Berman et al., 2009, 2017; Berman and Wurtz, 2010, 2011) that may contribute to mislocalization, then it is more likely to record from oblique rather than horizontal saccade representations. This is also true when probing the representations of brief perisaccadic flashes themselves. Therefore, and given the dependence of perisaccadic mislocalization strength on saccade direction (Grujic et al., 2018), true translation of the classic human mislocalization studies to neurophysiological experiments would only be possible with a general, and robust, two-dimensional mislocalization paradigm in monkeys. This is what we document here.

## Materials and Methods

### Experimental animals and ethical approvals

We collected data from three adult, male rhesus macaque monkeys (*Macaca mulatta*). The monkeys (A, F, and M) were aged 11–15 years, and they weighed 10–13 kg.

All experiments were approved by the ethics committees at the regional governmental offices of the city of Tübingen.

### Laboratory setup and animal procedures

The monkeys were seated in front of a brightly lit computer-controlled display, spanning ∼31° horizontally and 23° vertically. The display was at a distance of ∼72 cm from the animals. We tracked eye movements with high precision using the scleral search coil technique (Robinson, 1963; Fuchs and Robinson, 1966; Judge et al., 1980), and the experiment was controlled in real time using a modified version of PLDAPS (Eastman and Huk, 2012). Stimulus presentation and control were performed via the Psychophysics Toolbox (Brainard, 1997; Pelli, 1997; Kleiner et al., 2007), which provided exact time stamps at every frame update, ensuring accurate synchronization with the refresh cycle of the display. Prior tests with photodiodes (during the first few years of existence of the Hafed Laboratory) confirmed the accurate timings of the Psychophysics Toolbox, as expected given that the toolbox directly accesses the vertical blanking signal of the graphics card.

The animals were prepared for behavioral and neurophysiological experiments earlier (Tian et al., 2018; Buonocore et al., 2019; Skinner et al., 2019). This entailed a first surgery to implant a head fixation apparatus. During data collection, this apparatus was attached to a coupled device on the monkey chair in order to stabilize head position. Then, a later surgery was performed to implant a scleral search coil in one eye for eye movement tracking purposes.

### Experimental procedures

The main task involved generating a primary visually guided saccade, around which a very brief probe flash was presented; later on in the very same trial, the monkeys generated a “report” saccade aiming at where they remembered the perisaccadic flash to have appeared.

**Figure 1.**
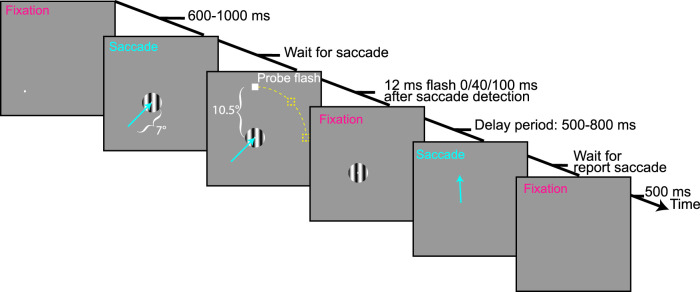
Perisaccadic visual mislocalization task for macaque monkeys. Each trial started with fixation of an eccentric spot (bottom-left corner of the display in the shown example). Upon removal of the spot, a saccade target appeared (Gabor grating), and the monkeys generated a visually guided saccade toward it (oblique cyan arrow). We detected saccade onset online, and we presented a brief, single-frame probe flash at one of the three possible delays from saccade detection. The probe flash could appear at one of the three possible locations on a virtual circle (dashed yellow curve and placeholders) around the saccade target: one directly ahead of the saccade target along the saccade vector direction and two at ±45° relative to the saccade vector (the shown example flash is at the 45° counterclockwise position). After an enforced delay, we removed the grating, and the monkeys reported their perceived flash location with a second, memory-guided, saccade. They had to maintain their gaze on the reported location for an additional delay time before being rewarded. In baseline trials, the probe flash appeared 100 ms after saccade detection and remained visible for 400 ms. Thus, it was clearly visible well after the end of the first saccade, resulting in an expected veridical percept.

Each trial (Fig. 1) started with an initial white fixation spot (of 79.9 cd/m^2^ luminance) presented over a uniform gray background (26.11 cd/m^2^). This illumination created a clear border along the edges of the display, providing a consistent visual reference frame throughout the experiments; thus, we were, in all likelihood, measuring “compression”-style mislocalization (Ross et al., 1997; Lappe et al., 2000). The fixation spot was displaced from display center (by 7°, except for the cardinal saccade directions of Monkey M, in which case it was by 5°), and it could appear in one of the eight (equally spaced) directions from this center. After 600–1,000 ms, the fixation spot disappeared, and a simultaneous saccade target appeared at the display center. This target consisted of a vertical Gabor grating of either 0.5 cycles/deg (cpd) or 8 cpd spatial frequency, and it had a radius of 1.5°. The grating had high contrast (100%), and it featured a white central marker surrounded by a small gray disc, as in our previous experiments (Baumann et al., 2023). This central marker aided in maintaining saccade accuracy and precision across trials having different image appearances of the saccade target. The onset of the grating was the cue to generate a visually guided saccade.

We monitored the monkeys’ eye position in real time and detected the onset of a saccade when the eye position deviated by 1.5° from the initial fixation spot. Following saccade detection, a probe flash was presented either immediately or with a delay of 40 or 100 ms (in subsequent post hoc analyses, we recalculated exact probe times; see below). The probe flash was displayed for a brief duration of ∼12 ms (one frame at a display refresh rate of 85 Hz) and was positioned 10.5° away from the center of the grating (7.5° for the slightly smaller cardinal saccades of Monkey M). The probe flash could appear either directly along the saccade vector direction (and farther away from the saccade target) or at an angle of ±45° relative to the saccade vector (again farther away than the saccade target location). Thus, there were three possible probe flash locations, all of which were expected to experience backward mislocalization for probe flash times very close to saccade onset, especially given the bright display and clear visual reference frames in the environment (Honda, 1999; Lappe et al., 2000).

After the monkeys made the visually guided saccade, with a maximum allowed reaction time of 500 ms, they maintained stable fixation on the saccade target for an additional 500–800 ms; this delay period started once the eye entered a predefined window around the saccade target. After the delay period, the grating disappeared, cueing the monkeys to make a memory-guided saccade “reporting” the remembered probe flash location. The monkeys were rewarded if they made the report saccade within <500 ms, and we allowed a radius of 8° tolerance window around the true flash location for the landing position of the eye. This large tolerance window was intentional because we wanted to avoid penalizing the monkeys for their mislocalized percepts; otherwise, the monkeys would have learned, over time, to constrain their report saccades to the regions gaining them rewards. On successful trials, the monkeys received juice rewards; on unsuccessful trials (e.g., late initial saccade or broken fixation), the trials were aborted, and juice rewards were withheld.

We also interleaved trials containing what we called a “baseline” condition. In this condition, which occurred in a total of 6.25% of all trials in a given session, the probe flash was always presented 100 ms after online saccade detection, and the probe also remained visible for a full 400 ms. That is, the probe was still clearly visible while the monkeys’ were in a stable fixation position on the grating center after the end of the primary saccade. This allowed assessing the monkeys’ eye movement behavior in the report saccade when no perceptual mislocalization was to be expected (of course, other small errors, such as those associated with memory-guided saccades, could still occur, as we also clarify in Results).

To avoid that the monkeys were solving the task by remembering absolute flash locations across trials, for example, by guessing their position relative to the edges of the display, we applied a global constant shift in all stimulus positions, which we changed randomly from trial to trial but which we kept constant for all stimulus events within a trial. Specifically, on every trial, the whole stimulus geometry (initial fixation position, final fixation position, and the virtual circle on which the probe flashes could occur) was shifted randomly by a constant known amount, within a range of ±0.5° both horizontally and vertically. In all analyses, all positions were rereferenced to the true saccade target location on any given trial. This is a similar approach to our recent human perisaccadic mislocalization experiments (Baumann et al., 2025).

Across data collection sessions, we varied the primary saccade direction, in order to demonstrate the two-dimensional flexibility of our paradigm. We selected eight distinct initial fixation positions on a virtual circle around the display center, allowing us to test eight different saccade vectors. Although our paradigm was designed to support any starting and end position in theory, we focused on these eight equally spaced directions for simplicity and for the opportunity to collect many trial repetitions of any given condition. In practice, we blocked trials of a given saccade direction in a given data collection session. This was done purely for pragmatic reasons. Specifically, we typically collected multiple sessions of data from each animal, and we wanted to have large numbers of trials for each saccade direction. Therefore, it was simpler to specify a given saccade direction for any given block that we ran. This is also the expected situation during neurophysiological experiments, in which a given saccade vector would be constant for a given recording site (e.g., in the superior colliculus). However, once the monkeys are proficient in this task, we believe that randomizing saccade directions within a single session would still be very easily performed by them (see our note on training trajectory below).

For Monkey M, as stated above, the cardinal saccades were only horizontal, and they had an amplitude of 5° (rather than 7°). Also, the probe flashes were 7.5° away from the saccade target in these cases (rather than 10.5). Thus, when summarizing this monkey's results, we separated the oblique conditions (with 7° saccades) from the cardinal ones (see Results). We did this in order to avoid potential confusion with respect to the quantitative amplitude of mislocalization for the two different saccade sizes that we tested in this animal. On the other hand, showing qualitatively similar results with different saccade sizes, both in the same monkey and also across animals (see Results), further confirms our interpretation of the robustness and flexibility of our visual mislocalization paradigm.

Finally, in the task above, the report by the animals was made through a memory-guided saccade to the “perceived” flash location. Memory-guided saccades have systematic (and variable) errors in their landing positions (White et al., 1994; Willeke et al., 2019), which can also depend on saccade direction (Hafed and Chen, 2016). However, as mentioned above, and as we also show in Results, we varied saccade directions across our experimental sessions, in order to convince ourselves that what we saw was true mislocalization (Grujic et al., 2018) and not only errors in memory-based reporting (Fig. 4 in Results). Moreover, the perisaccadic flash eccentricities were far enough from the saccade target to make the peak mislocalization errors significantly larger than any systematic or variable errors expected from memory-guided saccades (see Results).

We collected ∼350–500 successful trials per session from each monkey. In total, we analyzed 7,582, 16,005, and 20,902 successful and accepted trials for the saccade direction analysis, as well as 14,953, 23,830, and 23,371 successful and accepted trials for the saccade target appearance analysis from Monkeys A, F, and M, respectively (see below for our definitions of saccade direction data analysis and saccade target appearance data analysis).

### Training trajectory

Success in our task was built on a very simple training trajectory that each monkey underwent and that we describe here briefly. All three monkeys easily mastered the task.

All three monkeys were already experts in the individual task components before starting this project. Specifically, they knew how to perform both visually guided and memory-guided saccades. To get the monkeys to report flash position in our current mislocalization task, we first started with the visually guided saccade component of the paradigm; the monkeys simply made a saccade toward the grating image when they were instructed to do so (by the removal of the initial fixation spot and the appearance of the grating). They were also rewarded classically, immediately after removal of the grating image.

We then introduced a second delayed, visually guided saccade in every trial. Upon detection of the first saccade to the grating center, we presented the probe. However, instead of being flashed briefly, the probe remained visible until trial end. After the first saccade to the grating center, the monkeys performed a delayed saccade to the probe after grating removal. The reward now only came after a sequence of two instructed saccades (both visually guided). Since the monkeys were already proficient in delayed, visually guided saccades, this stage of training was achieved already from the first trial, allowing us to proceed.

We next introduced trials in which we presented the flash like in the main task above, except for two differences. First, the flash occurred always 100 ms after detecting the visually guided saccade. That is, we were sure that it should be minimally mislocalized since it appeared much later than the saccade time (Ross et al., 1997; Grujic et al., 2018). Second, the flash was presented for a long period of time (400 ms) on every trial (like in our baseline condition). This ensured that the flash was minimally mislocalized by the previous saccade and that it was still visible during stable fixation of the grating center. Upon removal of the grating, the monkeys made a relatively accurate memory-guided saccade toward the flash position, and we immediately rewarded them for having their gaze enter a relaxed virtual window of 8° radius around the true flash position. This made them quickly realize that the reward was now contingent on reporting the remembered flash position after completing their initial visually guided saccade.

We then gradually increased the period in which the monkeys had to maintain fixation on the remembered flash position before getting rewarded. This allowed them to maintain stable gaze position for a few hundred milliseconds before getting rewarded (Fig. 1). Thus, by now, the monkeys were proficient in performing an initial visually guided saccade to an image and then making a second memory-guided saccade toward the remembered flash location (with the flash itself being veridically perceived since it occurred long after saccade onset and also since it was visible for a long time). For one monkey (A), the animal had some difficulty transitioning to the memory-guided component of the task so far. Therefore, we introduced a few extra sessions in which the probe reappeared when the grating disappeared. This prompted the monkey to realize that the task was to focus on the remembered probe flash location.

When we were confident that the monkeys were performing this task with high fidelity, we then reduced the duration of the flash (down to a single display frame), and, finally, we started introducing the other flash times, in which we expected a large mislocalization error. As mentioned above, we maintained a relaxed virtual tolerance window radius around flash position, so that we did not penalize the monkeys for their mislocalized reports. All animals readily learned the task without much difficulties.

### Data analysis

#### Primary saccade processing

**Figure 2.**
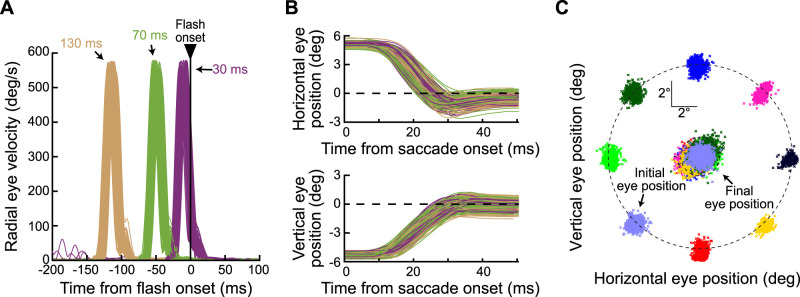
Controlling probe flash timing and saccade metrics and kinematics across conditions. ***A***, Radial eye velocity plots of all accepted primary saccades from an example session of Monkey A. All traces are aligned to the onset of the probe flash, revealing three distinct perisaccadic flash times, which we categorized by the three different colors. We expected progressively less visual mislocalization for longer times between the saccades and the probe flashes. Baseline trials are not included in this panel. ***B***, Horizontal (top) and vertical (bottom) eye position traces from one example saccade vector direction (up and to the left) out of the same example session shown in ***A***. Different colors show the different probe flash times, and the data suggest no systematic differences in the saccades across the flash times. ***C***, We also ensured that landing eye positions (central clouds of dots) overlapped for different saccade directions, again allowing us to investigate the influence of saccade direction on mislocalization strength with overlapping saccade properties (the kinematics were also matched as per the description in the text). The small black dashed circle indicates the extent of the grating toward which the primary instructed saccade was generated, and the big black dashed circle indicates the virtual circle along which the initial fixation positions were placed.

We detected all saccades (in post hoc analyses) using the methods that we described previously (Chen and Hafed, 2013). For the primary saccades, such detection allowed us to recalculate probe flash times relative to the actual saccade onset in the data. We found that the probe flashes in the experiment occurred at three distinct times from saccade onset, consistent with our experimental design. Based on the recalculations, we specifically classified the probe flash times into the following three categories: probes occurring ∼30 ms after primary saccade onset, probes occurring ∼70 ms after primary saccade onset, and probes occurring ∼130 ms after primary saccade onset. That is, the online saccade detection process described above took ∼30 ms to trigger the flashes during the data collection (expected given the dependence of our detection algorithm on eye position deviating sufficiently from initial fixation and also given the frame refresh rate of the display). Figure 2*A* shows an example of the relationship between primary saccade times and probe flash onset times from one example session of Monkey A. The figure plots radial eye velocity of the primary saccade (each curve is a saccade), with all trials aligned to probe flash onset time. There were three distinct time periods separating saccade and flash times across all trials.

Besides checking timing, we also checked primary saccade consistency across conditions. That is, we wanted to avoid the possibility that any differences in visual mislocalization that we uncovered (e.g., as a function of saccade direction) could be due to systematic differences in the actual primary saccade metrics and kinematics. Therefore, before analyzing the behavioral reports of the animals, we first filtered trials according to the eye movement data of the primary saccades. In one set of comparisons (see Results), we were interested in investigating the influences of primary saccade direction on perisaccadic visual mislocalization strength. Therefore, we ensured matched saccade vector and kinematic properties across the different directions of the primary saccades of our main task (Fig. 1). In another set of comparisons, we studied the influences of saccade target appearance on mislocalization strength; here, we matched the primary saccades across the different saccade target image types (low vs high spatial frequency).

For the vector and kinematic matching in the investigations of primary saccade direction influences, we binned all saccade main sequence data (Zuber et al., 1965; Bahill et al., 1975) into intervals of 0.5° in amplitude by 40°/s in peak velocity. We included in the analyses only those trials from the main sequence bins that had at least one trial from each of the eight saccade directions (four in the case of Monkey M's oblique saccade data; this monkey's cardinal saccades were analyzed separately due to their smaller size). This ensured that we could compare saccades across different directions having similar ranges of amplitudes and peak velocities.

We also noticed that Monkey M had a substantial difference in saccade peak velocity between rightward and leftward saccades (∼350 vs ∼550°/s, respectively). Therefore, for this monkey, we decided to report all analyses separately according to the hemifield of the saccade vector (see Results), meaning that we also separated the vector and kinematic matching procedures in this monkey for each hemifield individually. This way, we did not mask any possible quantitative effects of saccade peak velocity on mislocalization amplitude, but the results were always qualitatively the same across hemifields (and across animals).

Figure 2*B* shows, for the same monkey and session as in Figure 2*A*, example accepted saccades. Each panel shows either horizontal (top) or vertical (bottom) eye position aligned on saccade onset, and the different colors show the different probe flash time classifications of Figure 2*A*. Figure 2*C*, in turn, shows all start and end eye positions from all trials of all sessions of the same monkey. These trials passed the vector and kinematic matching (for the investigation of saccade direction influences on visual mislocalization), and they, therefore, went into further analysis.

For investigating the influences of saccade target appearance on mislocalization strength, we applied the same binning strategy above in order to achieve vector and kinematic matching, but we now compared saccades with either low or high spatial frequency Gabor gratings. We, thus, pooled across all saccade directions. We included only those trials from the main sequence bins that contained at least one trial repetition from each of the two saccade target image conditions (low and high spatial frequency) of each probe flash time. This ensured that saccades were matched for both metrics and kinematics across the different image conditions of the saccade target.

#### Establishing the existence of perisaccadic mislocalization

We next analyzed the report saccade endpoints, which constituted our experimental measure of perisaccadic visual mislocalization. We had three general classes of analyses in the study. First, we established that we were indeed measuring genuine mislocalization and not artifacts of either memory-guided saccade errors or our particular analysis choices. Second, we focused on the effects of saccade direction on mislocalization properties. Finally, we investigated the effects of saccade target appearance. We now describe the first class of analyses.

**Figure 3.**
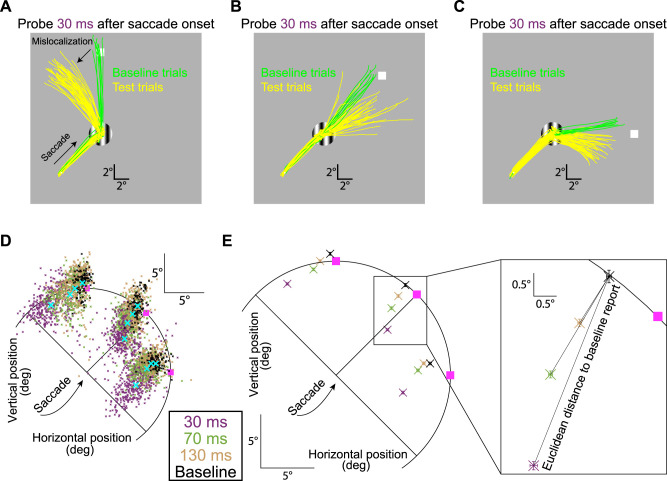
Two-dimensional perisaccadic “compression” in rhesus macaque monkeys. ***A***, Example eye position traces from Monkey F when generating an oblique (upward/rightward) primary saccade, followed by a second report saccade. Individual traces show the entire eye movement sequence per trial (see Materials and Methods), and we selected only trials with probe flashes directly above the saccade target location. The probe flash is schematized with a white square, but it was not actually visible at the time of the second saccade (the grating could also be different across trials; see Materials and Methods). Green traces show baseline trials in which the probe flash remained visible for a prolonged period after the end of the primary saccade, but it was still invisible at the report saccade time (see Materials and Methods). The monkey's report saccade was largely accurate, with a small systematic error expected from memory-guided eye movements. For perisaccadic flashes (yellow traces), there was a strong distortion in the opposite direction from the saccade vector (compare black oblique arrows). ***B***, ***C***, Example trials from the same monkey for two other flash locations. There was always reverse mislocalization. ***D***, Across all sessions from the same monkey, and for all probe flash times and the same oblique saccade direction, we plotted the two-dimensional reported locations. On baseline trials (black), the percepts were mostly veridical (with expected small, but systematic, errors associated with memory-guided saccades). For perisaccadic flashes (colored small dots), there was maximal mislocalization at 30 ms, with a gradual temporal recovery. This is consistent with human mislocalization time courses. The pink squares indicate true flash locations, and the cyan crosses show the means of each colored cluster (also presented with proper color coding in ***E***). ***E***, The main plot (left) shows mean and SEM summaries of the data of ***D***. For longer and longer times, gradual recovery toward baseline performance occurred at each probe flash location. On the right, we magnified the data for one probe flash location, to demonstrate our summary measures in subsequent figures: we characterized mislocalization strength by calculating the Euclidean distance between reports on mislocalization trials to reports on baseline trials (see Materials and Methods). In separate control analyses, we also checked the errors in baseline reports themselves (from true flash location).

To establish that we were measuring genuine mislocalization, we took the following measures, including control analyses to rule other potential explanations out. For each flash time and saccade direction, we measured the final eye position of the report saccade on every accepted trial, and we then plotted the raw two-dimensional data (Fig. 3*D* in Results) relative to the geometry of the saccade vector and probe flash location. We also collected summary statistics within each monkey. Specifically, for each probe flash location and time (for a given saccade direction), we calculated the mean and SEM of all reported probe flash locations. This allowed us to visualize the pattern of mislocalization (Fig. 3*E*, left, in Results).

We quantified mislocalization strength as follows. For each flash location, we used the baseline condition to know the average reported location by the monkey when perception was expected to be veridical. Then, for each trial of the same flash location but for one of the experimental perisaccadic flash times, we measured the Euclidean distance between the reported location of this trial and the mean reported location of the baseline condition (Fig. 3*E*, right, in Results). This allowed us to obtain average mislocalization across trials of a given flash time relative to any systematic error that might have existed in the report saccade in the absence of any perisaccadic mislocalization.

To explicitly confirm that we did indeed need to account for systematic errors in memory-guided saccades (White et al., 1994; Willeke et al., 2019), we also directly compared the error patterns between baseline and mislocalization trials relative to true flash location. For each trial (whether baseline or with a brief perisaccadic flash), we computed an error vector by connecting the veridical flash location to the reported location. We then calculated the angle of this vector relative to the primary saccade direction angle. If mislocalization errors were systematically related to the primary saccade direction, then their angular distributions were expected to be very tightly distributed ∼180° (i.e., backward mislocalization). However, on the baseline trials, if the errors were just systematic errors devoid of any perisaccadic mislocalization, then they were not expected to depend on the primary saccade direction; thus, the angle distributions relative to the primary saccade direction were expected to span 360°.

We also confirmed that baseline reports were always more veridical than the reports for perisaccadic flashes, and we verified that differences in baseline errors (e.g., as a function of saccade direction) were not sufficient to explain the differences in peak mislocalization effects that we reported in Results.

Having established that the baseline trials were the proper reference measure for estimating veridical percepts, we next considered whether memory decay could account for the larger localization errors observed in perisaccadic trials than in baseline trials (which we report in Results). Specifically, the longer flash durations in our baseline trials (relative to perisaccadic test trials) resulted in shorter delays between the probe flash offset time and the go signal for the animal to report the probe flash location. To confirm that this was not sufficient to explain the differences in error magnitudes between perisaccadic and baseline trials, we explicitly related localization error amplitude in baseline trials to delay period duration. We binned delay period durations into 30 ms bins, and we plotted the baseline report error amplitudes as a function of these bins. This allowed us to assess whether error amplitudes on perisaccadic probe flash trials were simple extrapolations of memory decay effects.

We then examined the relationship between mislocalization strength and flash onset time relative to saccade offset, by implementing a more fine-grained temporal binning approach. Within each of the three original coarse flash time conditions, we introduced additional temporal bins to increase resolution. This finer binning was made possible by the natural variability in saccade durations across trials, and it allowed us to confirm that we were likely not observing a biphasic mislocalization pattern as might be expected from pure darkness conditions (Honda, 1989, 1991, 1993, 1999).

To ensure that the observed mislocalization effects were also not merely a consequence of differences in eye position during (intrasaccadic and postsaccadic) probe flash presentations, we assessed the relationship between eye position at the time of the probe flash onset and the mislocalization strength. For each monkey and each probe flash time condition, we binned the mislocalization strength according to the distance between the eye position and the saccade target at the moment of the probe flash presentation. Bins were set at intervals of 0.2°. Eye positions were assigned negative values when the eye was located between the initial fixation point and the saccade target, and positive values indicated overshoots beyond the saccade target. This analysis allowed us to confirm that mislocalization magnitudes depended on flash times from primary saccade onset rather than on the exact eye position at probe flash occurrence (see Results).

On a related note, we next validated our expectation that differences in mislocalization strengths across probe flash presentation times were not driven by the presence of corrective saccades during the delay periods. We analyzed the temporal distribution of corrective saccades relative to the initial saccade onset. We aligned corrective saccade onset time to the initial saccade onset time, in order to assess these saccades’ timing across different probe flash time conditions.

#### Exploring the effects of saccade direction on perisaccadic mislocalization

After being convinced that we were measuring genuine perisaccadic mislocalization, we proceeded to summarizing the effects of saccade direction on visual mislocalization strength. In one analysis, we averaged the Euclidean distance measure described above across all trials that involved either upward or downward saccades for each probe flash time, respectively. Upward saccades in this kind of analysis included oblique upward/rightward and oblique upward/leftward saccades, in addition to purely vertical upward saccades. Similarly, downward saccades also included the oblique saccades with a downward component. In this comparison, we averaged across all three probe flash locations of a given saccade direction. In related analyses, we also measured the mislocalization strength (Euclidean distance to the reports in the baseline condition) for each saccade direction separately. Again, we pooled all flash locations per saccade direction together in this analysis, but we always treated flash times separately. Of course, we had previously inspected all the raw data of individual flash locations (Figs. 3*D*, 5*A*,*B* in Results) to justify pooling them in our summary analyses.

We also further summarized the difference in mislocalization strength between upward and downward saccades in a given analysis. To do so, we calculated the mathematical difference (at any given flash time) between the Euclidean distance measure (to the respective baseline condition of each flash location) obtained for upward saccades and the Euclidean distance measure obtained for downward saccades. We then plotted this difference measure across flash times. Again, this is a similar approach to how we reported changes in mislocalization strength across different experimental conditions in our recent human work (Baumann et al., 2025).

To once more validate that our observations were not dependent on our use of the baseline reports, we also repeated our saccade direction comparisons but by now relating the mislocalization errors to the reports on the trials with flashes presented ∼130 ms after saccade onset (rather than the baseline trials). That is, we exploited the fact that perception recovered with long times after saccade onset and used the latest flash time as the condition with closest-to-veridical percepts. As expected, similar results were observed.

Since perisaccadic mislocalization for a given saccade direction can also have a two-dimensional landscape (Kaiser and Lappe, 2004; Grujic et al., 2018), we additionally investigated the vector components of mislocalization, similar to what we had done recently with humans (Baumann et al., 2025). We examined the component of localization error that was parallel to the saccade direction and the component that was orthogonal. This analysis followed the same approach as that described above (measuring Euclidean distances to baseline reports for the different saccade directions), except that we separated the parallel and orthogonal components of the mislocalization errors.

In a separate comparison, we focused solely on the trials with horizontal primary saccades. In this case, we compared the Euclidean distance measure for probe flash locations in the upper versus lower visual fields. Thus, for such horizontal saccades, we compared the mislocalization strength between the two oblique probe flash locations associated with these saccades (by definition of the task, one oblique probe flash location would have been in the upper visual field and ahead of the saccade target location, and the other would have been symmetrically in the lower visual field). We should also note here that even for vertical saccades, probe flashes ahead of the saccade target could be in either the upper or lower visual field (e.g., upper visual field for upward saccades and lower visual field for downward saccades). For oblique saccades, some flash locations were at the same vertical eccentricity as the saccade target, but they were still in the upper visual field presaccadically for upward oblique saccades and still in the lower visual field presaccadically when the oblique saccade was downward. Thus, it is likely that some of our saccade direction effects interacted with visual field effects, as was also previously observed in humans (Grujic et al., 2018).

#### Exploring the effects of saccade target appearance on perisaccadic mislocalization

We also performed an investigation of the influences of the saccade target appearance on perisaccadic visual mislocalization. To do so, we averaged the Euclidean distance measures for all saccade directions and probe flash locations but now separated trials based on the spatial frequency of the saccade target.

#### Statistical analyses

We statistically compared different conditions using a two-way ANOVA on the Euclidean distance measure, with the two factors of the ANOVA being probe flash time relative to saccade onset and either saccade direction, flash location, or saccade target appearance, depending on the particular comparison that we were making. We always performed these analyses within each monkey individually, to be able to compare the outcomes across the individual animals. Although we consistently presented Monkey M's leftward and rightward saccades separately in all figures, we did not distinguish between them in the statistical analyses, as this separation did not affect the statistical results. We also statistically compared baseline reports under different comparison conditions. For example, we checked whether the errors in baseline reports (relative to the true flash location) were different whether saccades were upward or downward, and we did so by performing (for each monkey) a *t* test. Details are provided in the relevant sections of Results, and the statistics were performed with IBM SPSS (Version 29.0).

## Results

We aimed to establish a robust and flexible visual mislocalization paradigm in rhesus macaque monkeys. We tested this paradigm on three animals, and they all exhibited clear perisaccadic mislocalization, with expected recovery time courses. These animals also all replicated prior human findings of stronger mislocalization with upward saccades (Grujic et al., 2018), as well as stronger mislocalization for upper visual field flashes (with horizontal saccades; Baumann et al., 2025). We also found subtle effects of saccade target image appearance on mislocalization strength (Baumann et al., 2025), but these effects were not as consistent as with humans, and they were also not as strong as the effects of saccade directions and flash locations in the same animals. We summarize all of these observations below.

### Monkeys reported perisaccadic flashes as being closer to the saccade target

We trained our monkeys to report the location of a brief (∼12 ms) probe flash presented at different perisaccadic time points (Figs. 1, 2). They did so by directing their gaze, via a memory-guided saccade, to the remembered flash location after an instruction to do so (Fig. 1). Note that such memory-guided reporting of percepts is present even in classic human invocations of this same task (Ross et al., 1997; Lappe et al., 2000). To confirm that there was indeed mislocalization, we interleaved baseline trials in which the flash was long lasting and thus clearly visible after primary saccade end (see Materials and Methods). Therefore, any errors in reporting for this baseline flash condition should have been restricted to only those errors (whether systematic or variable) associated with memory-based behavioral reports (White et al., 1994; Willeke et al., 2019, 2022), and not directly related to perisaccadic mislocalization.

As a first step in our analyses, we performed a detailed investigation of unsuccessful trials that were not analyzed for mislocalization reports. We did this in order to gain further insights into the monkeys’ task performance and potential strategies. Unsuccessful trials were defined as those in which the monkey correctly initiated the trial by performing the instructed primary saccade (thereby triggering the probe flash presentation) but subsequently failed to obtain the reward due to performance-related errors. Trials in which the monkey did not initiate the task at all (e.g., due to a lack of motivation, blinks, or eye position instabilities at the initial fixation spot) were excluded from this analysis. Across all conditions, all three monkeys successfully completed 84.4, 79.3, and 76.7% (Monkeys A, F, and M, respectively) of the initiated trials, confirming that the probe flash was clearly visible under all experimental conditions. The failure analysis revealed two primary categories of unsuccessful trials: (1) fixation breaks and (2) incorrect saccade landings. Fixation breaks were errors in which the monkey broke fixation either before the go signal for the report saccade or during the final fixation period after the end of the report saccade. The proportion of fixation breaks was 9% for Monkey A, 12.5% for Monkey F, and 10.7% for Monkey M. Incorrect saccade landings were errors in which the monkey executed the report saccade but failed to land within the predefined spatial window required for successful reporting. The rates of this error type were 6.6% (shortest flash delay, 4.9%; medium delay, 1%; longest delay, 0.5%; and baseline, 0.2%) for Monkey A, 8.2% (shortest delay, 5.7%; medium delay, 1.3%; longest delay, 0.8%; and baseline, 0.4%) for Monkey F, and 12.5% (shortest delay, 9%; medium delay, 2%; longest delay, 0.5%; and baseline, 1%) for Monkey M. Thus, landing errors were always more frequent for the shortest probe flash times relative to the primary saccade onset.

The increased error rates for short probe flash delays are already the first hint that the monkeys really did experience perisaccadic visual mislocalization. Specifically, based on human research, shorter delays between saccade onset and probe flash presentation are associated with the largest mislocalization effects. Given the fixed spatial tolerance windows that we employed for accepting correct responses, larger mislocalization errors increased the likelihood that the monkeys’ reported locations fell outside of these windows, leading to a higher task failure rate. In essence, when the perceived location of the probe was strongly shifted, the monkeys might have been aiming accurately based on their perception, but the degree of mislocalization caused their responses to be registered as failures. This observation encouraged us to further analyze the successful trials for hallmarks of perisaccadic mislocalization. All results that follow are now solely based on the successful and accepted trials.

Figure 3*A* shows example results from Monkey F when generating an oblique upward/rightward primary saccade. The probe flash location in this case was rotated 45° counterclockwise from the saccade vector direction. In the figure, the probe flash is indicated by a white square, but it was not actually visible at the time of the report saccade. Each trace in the figure shows eye position from one example trial, and different traces show different trials. We plotted example trials from the baseline condition in green, and example trials from a perisaccadic condition (the one with the probe flash occurring 30 ms after saccade onset) in yellow. As can be seen, the primary saccades (oblique upward/rightward movements and directed toward the grating center) were all the same whether the trials were baseline trials or perisaccadic flash trials; that is, the eye position traces nicely overlapped with each other. This is expected given our eye movement controls mentioned earlier (see Materials and Methods). Importantly, the report saccades were very different depending on the properties of perisaccadic visual stimulation. In the baseline trials, the report saccades had a veridical direction (upward in this case); for brief perisaccadic flashes occurring 30 ms after saccade onset, the reported locations were strongly shifted backward along the reverse direction of the primary saccade vector (compare the black oblique arrows). Note that the baseline saccades were still memory guided, just like the report saccades in the perisaccadic test trials. Therefore, the large difference between report saccades in the two conditions in Figure 3*A* was attributed to brief, perisaccadic visual stimulation in one of the conditions, and not due to the memory-guided nature of the report saccades. Also note that there was larger variance in the report saccades in the perisaccadic test condition; this is also true in human perisaccadic mislocalization studies (Baumann et al., 2025). Thus, this monkey experienced strong backward perisaccadic mislocalization for brief flashes presented 30 ms after saccade onset, and this mislocalization happened with an oblique (rather than cardinal) saccade.

We also checked the other probe flash locations associated with this same saccade direction. Example traces from the same monkey and session are shown in Figure 3, *B* and *C*. Once again, there was always reverse mislocalization opposite in direction from the saccade vector. Specifically, in Figure 3*B*, the probe flash was directly ahead of the saccade target, and the reported locations were generally in the same direction as the flash but closer to the saccade target location (mislocalized backward in direction). Similarly, with the probe flash location rotated clockwise relative to the saccade vector (Fig. 3*C*), the reported locations were now deviated both leftward and downward relative to the baseline reports (resulting in a mislocalization vector directly opposite the upward and rightward vector of the primary saccade). Thus, the mislocalization that this monkey experienced perisaccadically was always backward relative to the saccade vector direction, regardless of flash location.

**Figure 4.**
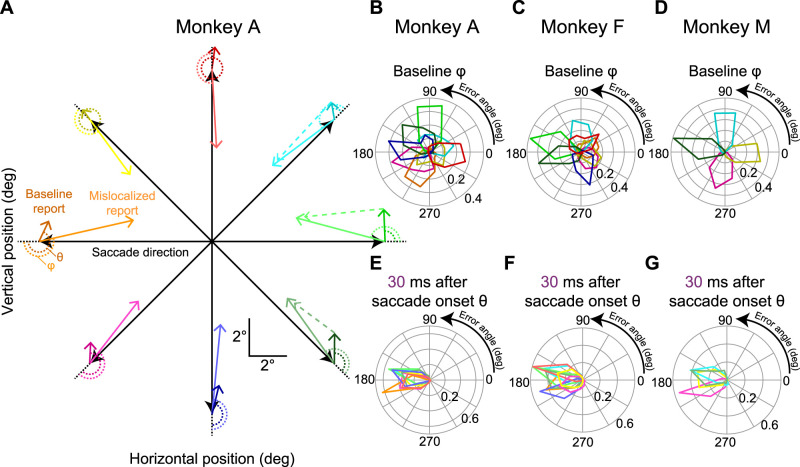
Comparison of error vector patterns between baseline and perisaccadic mislocalization trials. ***A***, Visualization of error vectors for Monkey A during the first flash time condition (30 ms after saccade onset), when mislocalization errors were most pronounced. Each colored vector connects the veridical flash location to the reported location, illustrating both baseline (darker-colored arrows) and mislocalization (brighter-colored, solid arrows) error vectors relative to the primary saccade direction (black arrows). Baseline errors consistently showed a small upward bias, independent of saccade direction. In contrast, mislocalization errors were systematically oriented in the direction opposite to the saccade vector. Dashed arrows indicate the direction of the mislocalization vector relative to the baseline report endpoint for selected saccade directions, demonstrating an even stronger 180° relationship with the primary saccade direction (other dashed vectors were not shown just to avoid clutter). ***B–D***, Polar histograms of angular errors (ϕ, as defined in ***A***) for baseline error vectors across all primary saccade directions for Monkeys A, F, and M. Baseline errors showed a broad distribution spanning all 360°, highlighting their independence from the saccade direction. Moreover, for each saccade direction, the baseline errors were clustered together, suggesting a single systematic baseline error direction independent of primary saccade direction (as in ***A***). ***E–G***, Polar histograms of angular errors (θ, as defined in ***A***) for mislocalization error vectors for Monkeys A, F, and M. Mislocalization errors were tightly clustered ∼180° relative to the saccade direction, indicating a robust and systematic divergence from baseline error patterns. Extended Data Figure 4**-**1 shows the relationship between baseline localization errors and the delay period preceding report saccade execution. Extended Data Figure 4**-**2 illustrates the relationship between mislocalization strengths and flash onset times relative to saccade offset, providing a more detailed time course depiction of perisaccadic mislocalization. Extended Data Figure 4**-**3 demonstrates a lack of influence of eye position at the time of the probe flash presentation on mislocalization strength. Extended Data Figure 4**-**4 shows the timing of corrective saccades relative to initial saccade onset across different probe flash presentation times.

Note also that there were systematic errors in the baseline report saccades themselves in Figure 3*A–C*, which are expected (White et al., 1994; Willeke et al., 2019). For example, in Figure 3*C*, a pure rightward report saccade was associated with a slight upward bias in eye position endpoints, along with saccade hypometry (White et al., 1994; Willeke et al., 2019, 2022). However, the perisaccadic mislocalization errors were always larger than these baseline report errors. Moreover, the perisaccadic mislocalization errors had a clear systematic relationship in direction relative to the saccade vector direction, as we also describe in more detail below. Thus, the perisaccadic mislocalization effects of Figure 3*A–C* were not fully explained by systematic errors associated with the reporting process itself (also see Fig. 4 below for a more detailed analysis of baseline reports).

10.1523/ENEURO.0547-24.2025.f4-1 Figure 4-1 **Relationship between delay period duration and baseline localization error.** Euclidean distance between the reported location and the actual probe flash position in baseline trials as a function of delay duration between primary saccade offset and the go signal for the report saccade. Each data point represents mean localization error per time bin, with error bars indicating SEM. The dashed purple lines indicate the peak mislocalization values for the individual monkeys, providing a reference for the maximal observed perceptual distortions in the perisaccadic period. Even with extrapolation for much longer delays, the baseline report errors were much smaller than actual perisaccadic mislocalization strengths. Download Figure 4-1, TIF file.

10.1523/ENEURO.0547-24.2025.f4-2 Figure 4-2 **Time course of perisaccadic mislocalization relative to saccade offset. (A-C)** Euclidean distance to the baseline report as a function of probe flash onset time relative to saccade offset for each monkey (red). Mislocalization gradually decreased with increasing delay after saccade offset, indicating a recovery of veridical localization over time. Error bars represent SEM. The faint histograms in the background show the actual underlying probe flash times; note that we did not plot red data points if there were fewer than 100 observations in any histogram bin. Download Figure 4-2, TIF file.

10.1523/ENEURO.0547-24.2025.f4-3 Figure 4-3 **Eye position at the time of probe flash presentation and its relationship to mislocalization strength. (A-C)** Euclidean distance (red) to the baseline report as a function of eye position at the time of probe flash presentation for probe flashes occurring 30  ms after saccade onset for the individual monkeys. **(D-F)** The same relationship for probe flashes occurring 70  ms after saccade onset. **(G-I)** The same analysis for probe flashes occurring 130  ms after saccade onset. In each case, the faint histograms in the background show the bins in which we had eye position data to document. Across all three monkeys and all time points, no systematic relationship was found between eye position and mislocalization strength, confirming that localization errors were primarily driven by the timing of the flash rather than by momentary gaze position. Indeed, for each monkey, the mislocalization strength decreased much more strongly from the top to bottom row than within each row as a function of eye position. Download Figure 4-3, TIF file.

10.1523/ENEURO.0547-24.2025.f4-4 Figure 4-4 **Corrective saccades were too late to account for mislocalization effects. (A-C)** The temporal distribution of corrective saccades relative to the initial saccade onset for each monkey. The number of corrective saccades is plotted over time, aligned to the initial saccade onset. The different probe flash timings (30  ms, 70  ms, and 130  ms after saccade onset) are color-coded. The majority of corrective saccades occurred after probe flash presentation, confirming that they did not influence perisaccadic mislocalization. A slight delay in corrective saccades for late probe flash presentations (130  ms) suggests transient saccadic inhibition, supporting the notion that the monkeys attended to the probe flash. The vertical black lines with surrounding dashed lines indicate the mean and SEM times of saccade offset. Download Figure 4-4, TIF file.

Across all trials and all probe flash times and locations for the same oblique saccade direction, we found systematic mislocalization that gradually recovered with time from saccade onset. This is shown in Figure 3*D*. Different colors indicate the different probe flash times, and individual small data points are individual trials (the cyan crosses show the means of each cluster). There was gradual recovery in mislocalization toward the baseline report locations when the probe flash time increased. This systematic trend can also be seen in Figure 3*E* (left), in which we plotted the mean and SEM summary statistics of the same data. In the same figure (Fig. 3*E*, right), we also explained our measure of mislocalization strength that we used in subsequent figures. Specifically, mislocalization strength was defined as the Euclidean distance to the baseline report location for each probe flash position (see Materials and Methods).

That the baseline reports were a proper reference point for canceling systematic errors of memory-guided saccades became even more clear to us when we related these baseline reports to individual saccade directions. Specifically, we compared error patterns in baseline trials with those observed during perisaccadic mislocalization (for the first flash time in which mislocalization was expected to be strongest from Fig. 3). The results of this analysis are presented in Figure 4. Figure 4*A* shows (for Monkey A) each primary saccade direction as a black arrow. For each such direction, we also represented the vector of the average baseline report error as a dark-colored arrow and the vector of mislocalization (for the shortest flash time) as a lighter-colored arrow of the same general hue. As can be seen, mislocalization vectors were always opposite the saccade vector, but baseline error vectors were always of the same size and direction independent of primary saccade direction. Consistent with this, measuring the angle of the baseline error vector relative to primary saccade direction resulted in distributions spanning 360° (Fig. 4*B*–*D*, with each panel showing each monkey). On the other hand, measuring the angle of the mislocalization report vectors relative to primary saccade direction always revealed a 180° relationship (Fig. 4*E–G*). Thus, our mislocalization reports (Figs. 3, 4) were very distinct from baseline errors. Most importantly, closer inspection of Figure 4*A* revealed that the baseline errors were (expectedly) always present, even in the mislocalization reports themselves. For example, for three sample primary saccade directions (those with a rightward component in the figure), when we corrected for the systematic baseline report vector error, mislocalization reports were better aligned with the 180° axis relative to the primary saccade direction than before. This makes perfect sense: if memory-guided saccades have systematic errors, then these systematic errors will still be present on brief probe flash trials. Thus, it was a valid choice for us to use baseline reports as the reference for veridical percepts in our analyses.

### The mislocalizations were not explained by factors related to the reporting mechanism or the properties of the primary saccade

Our results so far suggest that we replicated human evidence of reverse perisaccadic visual mislocalization. Importantly, we did so for a nonhorizontal saccade direction (Grujic et al., 2018), paving the way for generalized use of this paradigm in neurophysiological experiments. However, before diving deeper into further properties of perisaccadic visual mislocalization in macaque monkeys, we first ruled out some additional caveats that might come up, especially with respect to our specific reporting mechanism (memory-guided saccades).

First, our baseline trials had a particularly short time between probe flash onset time and the go signal for reporting (due to the long duration of the probe flash on these trials; Materials and Methods). Thus, it might be wondered whether our larger mislocalization effects in Figures 3 and 4 (for the nonbaseline trials) were due to decays in working memory associated with longer wait times on perisaccadic probe flash trials. To assess this, we analyzed the relationship between delay period duration after probe flash offset and localization accuracy on the baseline trials (Extended Data Fig. 4-1). Even though there was a very subtle increase in localization error with longer delay durations, the effect was minimal, and, even when extrapolated, it could not explain the mislocalization error amplitudes observed for the shortest flash onset times on the perisaccadic test trials. These findings align well with previous studies on memory-guided saccades, which demonstrated only minor (if any) memory decays over extended delays (White et al., 1994; Willeke et al., 2022). Thus, by including baseline trials in our analyses, we accounted for any general memory-related systematic errors, which were very temporally stable, ensuring that the reported mislocalization effects predominantly reflected genuine perisaccadic perceptual phenomena.

To better contextualize the temporal dynamics of mislocalization, we also examined the relationship between mislocalization strength and the onset time of the probe flashes relative to saccade offsets. Aligning the data on saccade offsets enabled more fine-grained temporal analyses due to saccade duration variability. Extended Data Figure 4-2 shows the time courses for individual monkeys, revealing a continuous decrease in mislocalization strength over time, indicative of a gradual recovery.

Because eye position could be variable at probe flash time, especially for intrasaccadic probes (shortest flash time), we also ruled out eye position effects as an explanation for the strong mislocalization results that we saw so far (Figs. 3, 4; Extended Data Fig. 4-2). We plotted the distance of the eye position from the saccade target at the moment of the probe flash presentation against mislocalization strength for each monkey and probe flash time (Extended Data Fig. 4-3). Across all three animals, no systematic relationship was observed between eye position at flash time and mislocalization strength. For instance, although the distributions of eye positions for probe flash times of 70 and 130 ms after saccade onset were very similar (as the saccade had already concluded for both flash times), a substantial difference in mislocalization strength persisted between these two conditions. This suggests that probe flash timing relative to the primary saccade exerted a much stronger influence on localization performance than the precise eye position at the time of flash presentation.

Finally, corrective saccades following the primary saccade also did not account for the observed differences in mislocalization strengths over time. In Extended Data Figure 4-4, we analyzed the temporal distribution of corrective saccades relative to the primary saccade onset. As expected from saccadic refractory periods, nearly all corrective saccades occurred after the probe flash presentations. Moreover, the timing of corrective saccades was highly similar for flash times of 30 and 70 ms after saccade onset. For trials with probe flashes presented 130 ms after saccade onset, corrective saccades were slightly delayed. This delay is due to saccadic inhibition, where the probe flash transiently suppresses the execution of corrective saccades when presented in close temporal proximity (Buonocore and Hafed, 2023). The presence of saccadic inhibition further supports the idea that the monkeys attended to the probe flash (White and Rolfs, 2016).

### Perisaccadic mislocalization was strongest for upward saccades

Having convinced ourselves that we were indeed measuring genuine perisaccadic mislocalization, we were now in a position to explore further properties.

We first focused on the influence of saccade direction. In previous work, we found that backward mislocalization in humans was particularly strong for upward saccades (Grujic et al., 2018). If our current paradigm is robust, and if macaques are indeed a suitable animal model for exploring this property of perisaccadic mislocalization with neurophysiology, then we should also obtain stronger perisaccadic visual mislocalization in our animals for upward saccades. This was indeed the case.

**Figure 5.**
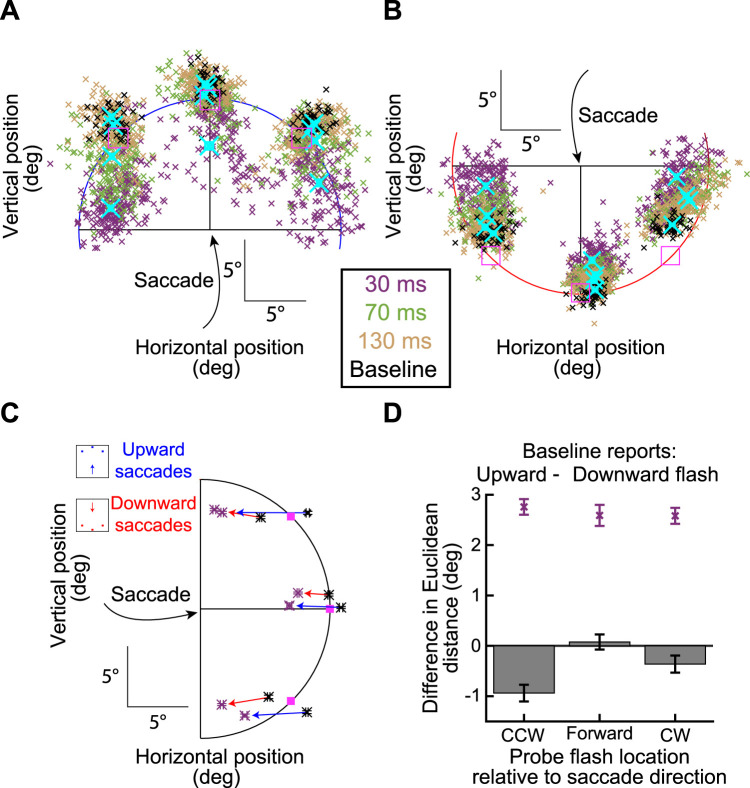
Stronger perisaccadic mislocalization for upward saccades. ***A***, Similar analysis to that in Figure 3*D* but for purely upward saccades in Monkey A. Reverse mislocalization can be seen, consistent with Figure 3. ***B***, In the same animal, the strength of reverse mislocalization was weaker for purely downward saccades. This can be seen by the smaller (than in ***A***) deviation in the reports for the 30 ms perisaccadic flashes from the baseline reports. Note that we showed the true flash location in ***A*** and ***B*** using outline pink squares in this case (unlike in Fig. 3), in order to avoid covering the data points. Otherwise, the figure is formatted like in Figure 3. ***C***, Comparison of the strength of mislocalization for the two saccade directions in ***A*** and ***B***. Here, we rotated the saccade vectors of ***A*** and ***B*** to a single schematic representation (shown here as a rightward saccade), like we did previously (Grujic et al., 2018). Purple symbols (along with SEM error bars) indicate reports with the 30 ms perisaccadic flashes, and black symbols (along with SEM error bars) indicate baseline reports. Pink squares indicate the true flash positions. Red and blue connecting arrows indicate whether the saccade was upward (blue) or downward (red). The blue arrows were always longer than the red arrows, indicating a stronger mislocalization (relative to baseline) for the upward than for the downward saccades. ***D***, Baseline reports exhibited minimal differences between upward and downward saccades for each flash location, highlighting that the stronger mislocalization for upward saccades relative to baseline reports (purple points) was not driven by differences in the baseline reports. For the individual probe flash locations, the difference in baseline reports between upward and downward saccades is shown as bars, and the difference in reports at peak mislocalization is shown as purple data points. All error bars denote SEM.

Consider the results of Figure 5, *A* and *B*, which are formatted similarly to Figure 3*D*. In Figure 5*A*, the animal (Monkey A) generated purely upward saccades. The mislocalization was backward relative to the saccade direction (also see Fig. 4), and it was stronger for smaller perisaccadic probe flash times than for later times, as expected. Importantly, the mislocalization seen in Figure 5*A* was substantially stronger than that observed for purely downward saccades in the same animal. Specifically, in Figure 5*B*, we plotted the data for purely downward saccades in the same format as in Figure 5*A*. There was still reverse mislocalization opposite the saccade direction, but the peak mislocalization strength (for 30 ms perisaccadic probe flash times) was significantly weaker than in Figure 5*A*. This fact is rendered clearer by noting Figure 5*C*. Here, we plotted the average (and SEM) reported locations for 30 ms perisaccadic probe flashes for the two saccade directions together in the same plot. To facilitate comparison of the effect sizes in the two cases, we rotated all data to a schematic representation in which the saccade vector was to the right, and the probe flashes were further ahead of it. We used exactly the same approach to visualize results from different saccade directions in our earlier human studies (Grujic et al., 2018). As can be seen, relative to the baseline reports of each condition (black symbols and colored connecting arrows), there was clearly stronger perisaccadic mislocalization (in the backward, reverse direction) for upward saccades. Quantitatively, for purely upward saccades, the average Euclidean distance measure across all three flash locations in this figure was 5.8°, whereas it was only 3.5° for purely downward saccades. Therefore, there was stronger mislocalization for the upward saccades.

Moreover, this difference in mislocalization strength between upward and downward saccades at peak mislocalization (30 ms flash times) was clearly larger than any systematic differences in the baseline report locations between upward and downward saccades: the Euclidean distance between the baseline report locations (black symbols) for purely upward and purely downward saccades in Figure 5*C* was only 0.51° (again averaging the respective distances across all three flash locations); this was smaller than the difference in mislocalization strengths between the upward and downward saccades. This result can be seen in Figure 5*D*, in which we plotted the difference in baseline reports for upward and downward saccades (for the individual flash locations) as bars; the difference at peak mislocalization between upward and downward saccades is shown as purple data points, confirming that the larger difference in mislocalization strengths between upward and downward saccades was not a consequence of equally large differences in baseline reports. Thus, there was indeed stronger perisaccadic visual mislocalization for upward saccades, replicating previous human observations (Grujic et al., 2018).

**Figure 6.**
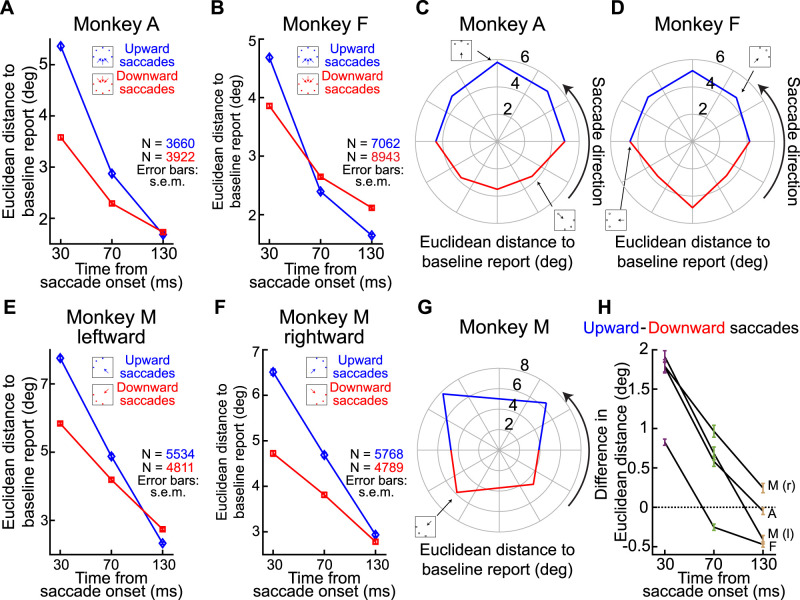
Consistently stronger perisaccadic mislocalization for upward saccades. ***A***, In Monkey A, we measured the Euclidean distance of perisaccadic reports (relative to baseline reports) as a function of flash time from saccade onset. The two curves show mislocalization strengths when considering only trials with either upward (blue) or downward (red) saccades. The inset icons clarify the saccade vectors included for each curve (see Materials and Methods), as well as their relevant flash locations (three per saccade direction). There was stronger mislocalization for upward saccades. ***B***, Similar results for Monkey F. This monkey also showed more rapid recovery dynamics, with time, for the upward saccades. ***C***, ***D***, Same data as in ***A*** and ***B*** but considering each saccade direction individually. In each plot, the angle indicates the direction of the saccade (see inset icon examples), and the radial value indicates the Euclidean distance measured for 30 ms flash times. There was stronger mislocalization for upward saccades. ***E***, ***F***, Similar analyses to ***A*** and ***B*** but for Monkey M. In this monkey, we separated saccades with a leftward (***E***) or rightward (***F***) component. In both panels, the monkey still exhibited stronger mislocalization for upward saccades. ***G***, Same as ***C*** and ***D*** but for Monkey M. Again, there was stronger mislocalization for upward saccades. Note that there was also stronger mislocalization for leftward saccades. These were the faster eye movements (∼550°/s vs ∼350°/s peak velocity), suggesting an interaction between saccade speed and mislocalization strength (Ostendorf et al., 2007). ***H***, We summarized the effects of saccade direction by calculating the difference in mislocalization strength between upward and downward saccades. For all monkeys, there was stronger mislocalization for upward saccades, especially at peak mislocalization times. Note that for Monkey M, we show two curves for either leftward (l) or rightward (r) saccades. Error bars indicate SEM.

We next summarized the above observations across all three animals. In Figure 6, *A* and *B*, we measured the Euclidean distance of flash localizations (Fig. 3*E*, right; see Materials and Methods) as a function of probe flash time from saccade onset. We did so separately for all upward saccades and for all downward saccades. In the two shown animals in these two panels, all upward saccades meant purely upward and two oblique upward saccade directions, and all downward saccades meant purely downward and two oblique downward saccade directions (Fig. 6*A*,*B*, inset schematics). We also included all flash locations per saccade direction. That is, for each saccade direction and saccade location, we collected the Euclidean distance, and we then averaged across all measurements. In both animals, at peak mislocalization (flashes occurring 30 ms after saccade onset in our experiments), there was stronger perisaccadic visual mislocalization for upward saccades, consistent with human observations (Grujic et al., 2018). We also reached the same conclusions when treating each saccade direction individually (Fig. 6*C*,*D*).

We also replicated these results in Monkey M. In this monkey, we separated the visual hemifields toward which the primary saccades were directed because this monkey had significantly slower saccades with a rightward component, making it difficult to match kinematics across hemifields (see Materials and Methods). The monkey again exhibited stronger perisaccadic visual mislocalization for upward saccades (Fig. 6*E*,*F*). This can also be seen in Figure 6*G*, with the added observation being that perisaccadic visual mislocalization was stronger for the faster leftward saccades. This is also consistent with human observations (Ostendorf et al., 2007).

Statistically, all of the above results were robust at the individual monkey level. We conducted a two-way ANOVA to examine the effects of saccade direction and probe time relative to saccade onset on the Euclidean distance to the baseline report. There was a significant main effect of saccade direction for Monkeys A (*F*_(1,7576)_ = 421.07; *p* < 0.001) and M (*F*_(1,20896)_ = 812.77; *p* < 0.001), indicating that the direction of the saccade had a significant impact on perisaccadic mislocalization. Monkey F did not show a significant main effect for saccade direction (*F*_(1,15999)_ = 1.92; *p* = 0.16), but this was only because this monkey exhibited faster recovery times than the other two monkeys (Fig. 6*B*). Indeed, this monkey, as well as the other two, showed a significant interaction between saccade direction and probe flash time relative to saccade onset (Monkey A, *F*_(2,7576)_ = 202.37; *p* < 0.001; Monkey F, *F*_(2,15999)_ = 265.66; *p* < 0.001; Monkey M, *F*_(2,20896)_ = 333.72; *p* < 0.001), indicating that the effect of saccade direction depended on the time point. There was also a significant main effect of probe time relative to saccade onset in all three monkeys (Monkey A, *F*_(2,7576)_ = 1,870.19; *p* < 0.001; Monkey F, *F*_(2,15999)_ = 3,371.03; *p* < 0.001; Monkey M, *F*_(2,20896)_ = 4,434.12; *p* < 0.001), suggesting that saccadic mislocalization varied across different time points (as expected). Post hoc comparisons using Bonferroni’s corrections for probe time relative to saccade onset revealed significant differences between all time points for all monkeys, with visual mislocalization decreasing progressively from 30 to 130 ms (*p* < 0.001). All of these statistical results are summarized graphically in Figure 6*H*.

We also quantified mislocalizations for probe flash presentations at 30 and 70 ms after saccade onset by measuring the Euclidean distance of these reports not to the baseline trials but to the reported locations of trials with probe flash presentations at 130 ms after saccade onset. For Monkey A, the average mislocalization error for upward saccades was 4.9° ± 0.06 SEM at 30 ms and 2.4° ± 0.04 SEM at 70 ms, while for downward saccades, the error was only 3.1° ± 0.04 SEM (30 ms) and 1.7° ± 0.03 SEM (70 ms). Monkey F [upward saccade, 3.8 ± 0.03° (30 ms); 1.8 ± 0.02° (70 ms); downward saccade, 2.7 ± 0.03° (30 ms); 1.6 ± 0.02° (70 ms)] and Monkey M [upward saccade (to the left), 6.6 ± 0.05° (30 ms); 3.8 ± 0.05° (70 ms); downward saccade (to the left), 4.5 ± 0.04° (30 ms); 3 ± 0.04° (70 ms); upward saccade (to the right), 5.6 ± 0.05° (30 ms); 3.4 ± 0.04° (70 ms); downward saccade (to the right), 4.3 ± 0.05° (30 ms); 3.1 ± 0.04° (70 ms)] showed a similar pattern.

**Figure 7.**
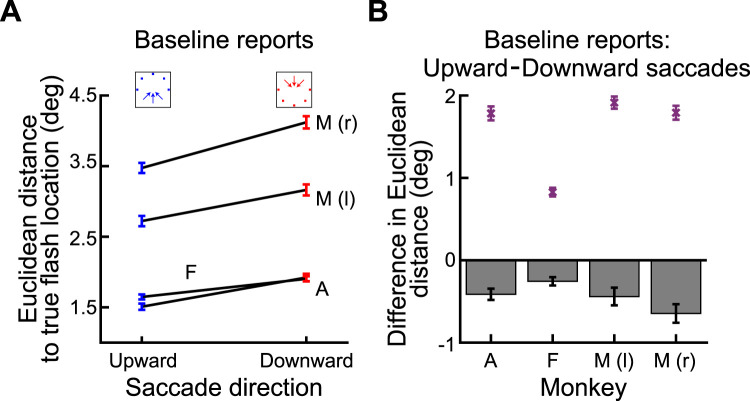
Different patterns of baseline report errors between upward and downward saccades than for mislocalization. ***A***, For each monkey, we measured the Euclidean distance between the baseline report saccade endpoints and the true flash locations in the data of Figure 6. We did this separately for either upward or downward saccades. Error bars denote SEM. Note how there was still some error from the true flash location in the baseline reports in which no mislocalization was to be anticipated (also visible in Figs. 3, 5). This is expected from memory-guided saccades (White et al., 1994; Willeke et al., 2019, 2022). However, such error was always smaller than peak mislocalization error (also visible in Figs. 3, 5). ***B***, For each monkey, we took the difference in the errors in ***A*** between upward and downward saccades (bar plots; similar to Fig. 5*D*). This difference revealed slightly larger errors for downward memory-guided saccades, consistent with earlier observations (Hafed and Chen, 2016). In contrast, the purple data points are the results from the time of peak mislocalization in Figure 6. There was a clearly larger difference between upward and downward saccades during peak perisaccadic visual mislocalization than during baseline reports (and also with an opposite sign). Therefore, the results of Figure 6 were not driven by differences in baseline report errors between upward and downward saccades.

Returning to our more robust measures relative to baseline reports, and like we did in Figure 5*D*, we also checked the differences in systematic errors in baseline reports themselves. Specifically, since memory-guided saccades of different directions can have different systematic error magnitudes (Hafed and Chen, 2016), we asked whether the differences in mislocalization strengths between upward and downward saccades that we saw in Figure 6 were entirely due to differences in baseline report errors. For each monkey, we measured the error (from true flash location) in the baseline report saccade as a function of saccade direction (Fig. 7*A*). We did this only for the baseline trials in which perception was expected to be veridical (see Materials and Methods). As anticipated (Figs. 3, 5), there were nonzero absolute errors in the baseline saccades. However, these errors were significantly smaller than the peak mislocalization effects (also seen in Figs. 3, 5). For example, Monkey F had a peak mislocalization amplitude of ∼4–5° (Fig. 6*B*), but only a baseline report error of ∼1.5–1.6°. Most importantly, in all animals, the difference in baseline report errors between upward and downward saccades (Fig. 7*B*), although significant (Monkey A, *t*_(647)_ = −6.04; *p* < 0.001; Monkey F, *t*_(1323)_ = −5.03; *p* < 0.001; and Monkey M, *t*_(689)_ = −4.11; *p* < 0.001 for leftward saccades and *t*_(704)_ = −5.78; *p* < 0.001 for rightward saccades; independent sample *t* tests), was of the opposite sign from the mislocalization effects comparing upward and downward saccades (Figs. 6, 7*B*, purple samples). Thus, stronger perisaccadic mislocalization for upward saccades was not explained by systematic differences in baseline report errors.

**Figure 8.**
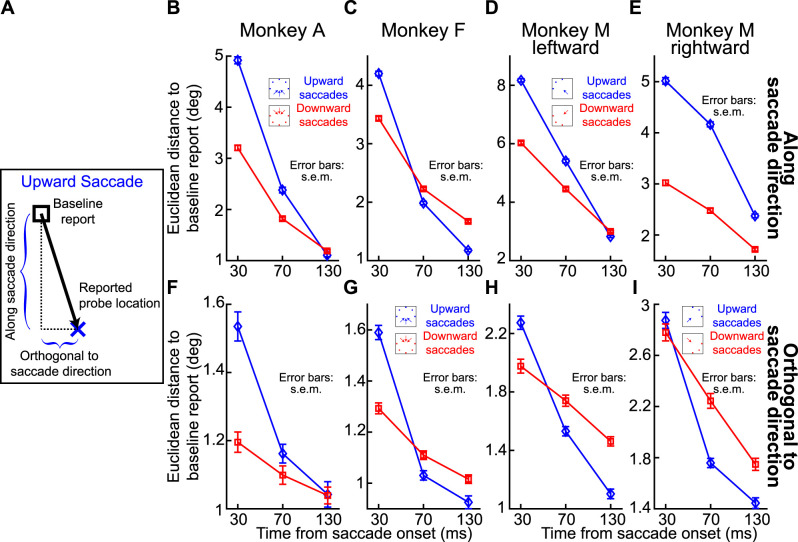
Two-dimensional perisaccadic mislocalization landscape in macaques. ***A***, Our analysis approach for assessing the strength of orthogonal mislocalization (Baumann et al., 2025). We measured the two components of mislocalization separately. One component was along the saccade direction, and one component was orthogonal to the saccade direction (Baumann et al., 2025). ***B*–*E***, Mislocalization along the saccade direction for upward (blue) and downward (red) saccades in the three monkeys (A, F, M). Note that we show rightward and leftward saccade directions separately for Monkey M. Error bars indicate SEM. There was consistently stronger mislocalization for upward saccades. ***F*–*I***, Localization error orthogonal to the saccade direction for the same conditions. Stronger mislocalization for upward saccades was still evident, with a pronounced effect along the saccade direction and a smaller but consistent error orthogonal to the saccade direction across all tested monkeys.

Finally, mislocalization is known to exhibit a two-dimensional landscape, with a component parallel to the saccade direction and another orthogonal to it (Fig. 8*A*). In Figure 8, stronger mislocalization for upward saccades was evident in both directional components. Both the larger mislocalization effect along the saccade direction (Fig. 8*B*–*E*) and the smaller localization error orthogonal to the saccade direction (Fig. 8*F*–*I*) varied depending on the saccade direction in all three tested monkeys.

Therefore, our results so far indicate that all three monkeys experienced perisaccadic visual mislocalization and that they all demonstrated an effect of saccade direction, consistent with human observations (Grujic et al., 2018). A quantitative comparison of effect magnitudes as a function of saccade speeds was also afforded to us by Monkey M, and the results were again consistent with human observations (Ostendorf et al., 2007). This reinforces our interpretation of the flexibility and robustness of our paradigm.

### Perisaccadic mislocalization was stronger for upper visual field flashes

**Figure 9.**
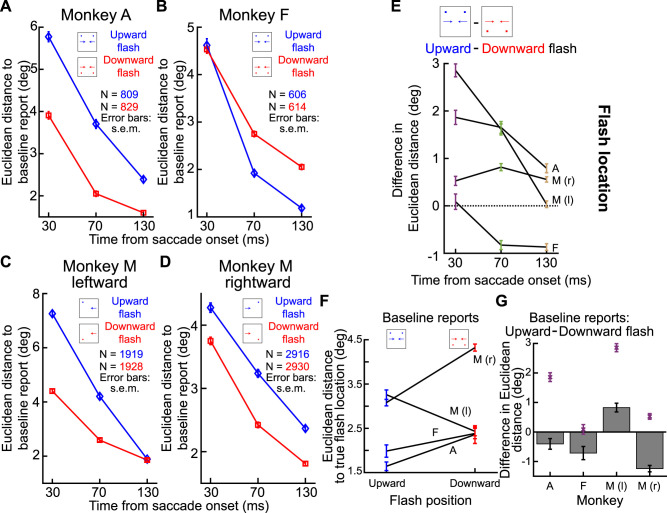
Stronger perisaccadic visual mislocalization for upper visual field probe flashes and horizontal saccades. ***A***, For Monkey A, we only considered horizontal primary saccade directions. We then compared mislocalization strength (Euclidean distance to baseline reports) for probe flashes above or below the saccadic vector (see Materials and Methods and inset schematic). There was stronger mislocalization for upper visual field flashes, like in humans (Baumann et al., 2025). ***B***, In Monkey F, the effect at peak mislocalization (flashes 30 ms from saccade onset) was much weaker than in Monkey A but with a similar trend. This monkey also had rapid time dynamics, like in Figure 6, causing a reversal at longer times. ***C***, ***D***, Monkey M replicated the results of Monkey A regardless of the direction of the horizontal saccades. ***E***, Summary of the results in ***A*–*D*** shown here as the difference in mislocalization strength between upper and lower visual field flash locations and as a function of flash time (like in Fig. 6*H*). ***F***, Errors in baseline reports from true flash location, like in Figure 7*A*. Here, we plotted these errors for either the upward or downward flash conditions. The errors were always smaller in magnitude than the peak mislocalization effects in ***A*–*E*** (consistent with Figs. 3, 5). ***G***, More importantly, the difference in these errors between upward and downward flashes did not explain the differences in peak mislocalization. This panel is formatted similarly to Figure 7*B*: the bars denote the differences in baseline report errors between upper and lower visual field flashes, and the purple symbols show the peak mislocalization differences from ***A*** to ***E***. Differences in baseline reports did not explain differences in peak mislocalization strengths (except for Monkey F where there were already no differences in mislocalization strength). Error bars indicate SEM.

We next explored the effect of visual field location of the flashes relative to horizontal saccades. In humans, we recently found that, with such horizontal saccades, reverse mislocalization was stronger for upper visual field flashes (ahead of the saccade target location) than for symmetrically located lower visual field ones (Baumann et al., 2025). This was also the case in our monkeys. Specifically, in Figure 9*A*–*D*, we analyzed mislocalization strength for only horizontal saccades in each monkey. This time, we separated the trials based on whether the perisaccadic flash location was above or below the horizon. That is, if the saccade was to the right, our paradigm included three potential flashes: one directly ahead of the saccade target (still on the horizontal axis) and two oblique ones (Fig. 1; see Materials and Methods). The two oblique flashes were, by definition, either in the upper or lower visual field relative to the saccade direction, and we compared these two oblique flash positions in the current analysis. The results demonstrated that there was indeed generally stronger mislocalization associated with upper rather than lower visual field flashes.

Statistically, we performed a two-way ANOVA to evaluate the effects of probe location and probe time relative to horizontal saccade onset on the Euclidean distance to the baseline report. There was a significant main effect of probe location for all three monkeys (Monkey A, *F*_(1,1632)_ = 410.01; *p* < 0.001; Monkey F, *F*_(1,1214)_ = 65.2; *p* < 0.001; Monkey M, *F*_(1,9687)_ = 626.66; *p* < 0.001) indicating that the probe location had an impact on the strength of perisaccadic visual mislocalization. The main effect of probe time relative to saccade onset was also significant (Monkey A, *F*_(2,1632)_ = 552.54; *p* < 0.001; Monkey F, *F*_(2,1214)_ = 714.44; *p* < 0.001; Monkey M, *F*_(2,9687)_ = 1,652.95; *p* < 0.001), suggesting that perisaccadic mislocalization varied across different time points. Finally, the interaction between probe location and probe time relative to saccade onset was also significant for all three monkeys (Monkey A, *F*_(2,1632)_ = 21.43; *p* < 0.001; Monkey F, *F*_(2,1214)_ = 21.93; *p* < 0.001; Monkey M, *F*_(2,9687)_ = 70.51; *p* < 0.001), indicating that the effect of probe location was dependent on flash time. These results are summarized in Figure 9*E* showing difference measures similar to the ones that we used earlier for summarizing saccade direction effects.

We also checked whether systematic differences (between upper and lower visual field flashes) in baseline report errors accounted for the above results. Specifically, just like in Figure 7*A*, we plotted the Euclidean distance between baseline reports and true flash location for the upward and downward flash conditions of this analysis. The results are shown in Figure 9*F*. Consistent with Figure 7*A*, the absolute errors in baseline reports were smaller than the peak mislocalization effects; this is also consistent with the raw example data shown in Figures 3 and 5, demonstrating clear recovery with time from saccade onset (toward the baseline report locations). Importantly, the differences in baseline report errors between upward and downward flashes were not correlated with the differences in peak mislocalization strength (Fig. 9*G*). Thus, the effects of upper and lower visual field flashes in association with horizontal saccades were likely perisaccadic and not an artifact of report saccade errors.

### Perisaccadic mislocalization strength was subtly modulated by the saccade target appearance

**Figure 10.**
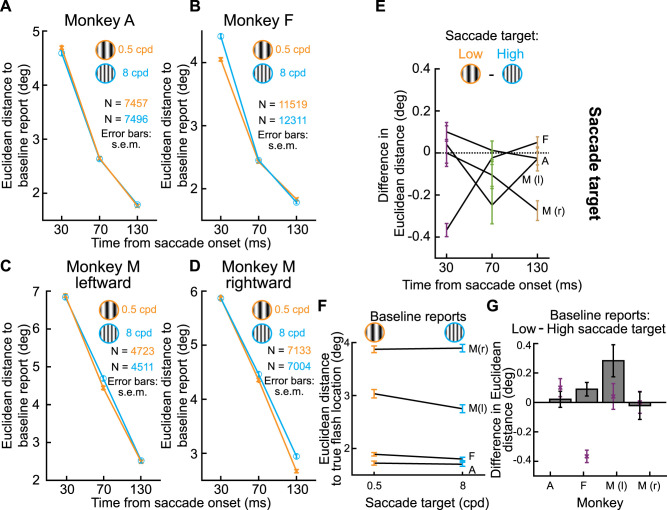
Inconsistent effects with respect to saccade target appearance. This figure is formatted identically to Figure 9. Here, the comparison was between the different saccade target appearances. Therefore, we pooled across saccades of all directions (see Materials and Methods) and only separated trials based on the appearance of the saccade target. There were subtle differences (***A*–*E***), but the differences were similar to the differences in baseline report saccades in the absence of perceptual mislocalization (***F***, ***G***). Thus, the image dependence effect in perisaccadic mislocalization (Baumann et al., 2025) was likely masked by systematic errors in report saccades. All other conventions are similar to Figure 9.

Finally, we explored whether mislocalization strength depended on the visual appearance of the saccade target (see Materials and Methods). In two out of the three monkeys, there was a very subtle difference in mislocalization strength as a function of saccade target appearance; however, this dependence was not the same in both animals. Consider, for example, Figure 10*A*. The figure shows slightly stronger mislocalization for the low spatial frequency grating as the saccade target. However, Monkey F showed the opposite effect (Fig. 10*B*). Figure 10, *C* and *D*, shows the data of Monkey M, showing no image dependency for the time bin with peak mislocalization and neither for leftward nor for rightward saccades. These findings for the image dependence are smaller than the dependence for saccade direction.

Statistically, we performed a two-way ANOVA to investigate the effects of saccade target appearance and probe time relative to saccade onset on the Euclidean distance to the baseline report. There was a significant main effect of saccade target appearance for two of the three monkeys (Monkey A, *F*_(1,14947)_ = 1.15; *p* = 0.28: Monkey F, *F*_(1,23824)_ = 33.05; *p* < 0.001; Monkey M, *F*_(1,21454)_ = 20.32; *p* < 0.001) indicating that the saccade target had an impact on the strength of perisaccadic mislocalization. However, Monkeys M’s and F’s results were not consistent with the previous human results: these monkeys exhibited stronger mislocalization for a high spatial frequency grating. As expected, there was also a significant main effect of probe time relative to saccade onset (Monkey A, *F*_(2,14947)_ = 3,867.51; *p* < 0.001; Monkey F, *F*_(2,23824)_ = 5,413.83; *p* < 0.001; Monkey M, *F*_(2,21454)_ = 4,889.48; *p* < 0.001), suggesting that saccadic mislocalization varied across different time points. Additionally, the interaction between saccade target appearance and probe time relative to saccade onset was significant for all three monkeys (Monkey A, *F*_(2,14947)_ = 1.87; *p* < 0.001; Monkey F, *F*_(2,23824)_ = 42.36; *p* < 0.001; Monkey M, *F*_(2,21454)_ = 4.77; *p* < 0.001).

Thus, we cannot confidently conclude that there was an image dependence of mislocalization strength in our monkeys. This is likely caused, at least in part, by the differences in the ways monkeys and humans reported their percepts (see Discussion). Indeed, analyzing the baseline report errors was particularly revealing (Fig. 10*F*,*G*): the difference in magnitudes of baseline report errors between the different target image appearances (Fig. 10*G*) was of the same order of magnitude as the difference in the peak mislocalization strengths for the different saccade target images. This suggests that a possible image dependence of perisaccadic mislocalization in our monkeys might have been masked by errors in the memory-guided report saccades.

## Discussion

We established a robust paradigm with which we could probe perisaccadic mislocalization (with clear visual references in the environment) in monkeys. Importantly, our paradigm allows mapping the two-dimensional landscape of mislocalization, and it can also be used with arbitrary saccade directions. This latter property enabled us to replicate the observation in humans (Grujic et al., 2018) that backward perisaccadic “compression” of flashes ahead of the saccade target is strongest for upward saccades. This dependency of mislocalization on saccade direction was also visible in both directional components, along and orthogonal to the saccade direction. Moreover, we replicated the human observation (Baumann et al., 2025) of stronger backward mislocalization for upper visual field stimuli during horizontal saccades.

Our results can be compared with human psychophysics conducted under similar visual reference conditions. Specifically, a biphasic perisaccadic mislocalization pattern, as reported by Honda (1991, 1993), is a well established phenomenon observed primarily under complete darkness. In this case, when flashes (regardless of their location) are presented immediately before or at the onset of a saccade, mislocalization typically occurs in the same direction as the saccade. Conversely, when flashes are presented at the end or immediately after the saccade, the perceived location of the flash shifts in the direction opposite to the saccade direction, resulting in a characteristic forward and then backward pattern of mislocalization​. However, the presence of visual references significantly alters this effect. For example, Honda (1999) demonstrated that introducing even minimal visual context, such as a faint frame pattern or a dimly illuminated structured background, substantially reduced mislocalization and disrupted the classic biphasic pattern. Under these conditions, mislocalization was more akin to a “compression” effect, where the perceptual error depended on the flash position relative to the saccade target rather than purely on the temporal relationship to the saccade onset. That is, whether before or after the saccade, flashes nearer to the initial fixation position than the saccade target were generally mislocalized forward in the same direction as the saccade, whereas flashes farther than the saccade target underwent backward mislocalization. These findings align well with the results of other studies (Ross et al., 1997; Lappe et al., 2000), which also showed that structured visual environments promoted “compression”-like mislocalization patterns. Since we included a stable visual reference (the saccade target) and bright display conditions, our results are consistent with this framework. Of course, in future work, we can confirm this explicitly by testing probe flash locations in between the initial and final saccade fixation points (Grujic et al., 2018). Measuring such probe locations would allow us to assess the spatial profile of mislocalization more comprehensively. Specifically, if probes placed between the initial fixation point and the saccade target were to be mislocalized in the forward direction relative to the saccade, then this would indicate a compressive mechanism. In contrast, if all probe locations underwent mislocalizations that resembled the forward then backward distortions described in Honda (1991, 1993), then this would suggest the presence of a shift component.

Our work builds on previous demonstrations that perisaccadic visual mislocalization can be studied in macaques (Dassonville et al., 1992; Jeffries et al., 2007; Klingenhoefer and Krekelberg, 2017; Weng et al., 2024). Our work also relates to interesting monkey variants of the classic double-step saccade task (Hallett and Lightstone, 1976; Becker and Jurgens, 1979; Ottes et al., 1984; Vliegen et al., 2005), in which the target of the second saccade in the double-step sequence was presented during the progression of the first eye movement (and thus could be susceptible to some mislocalization; Van Grootel et al., 2012). However, in the context of visual mislocalization studies, our approach adds paradigm flexibility that we believe is necessary for further success in porting these behavioral measures of perisaccadic mislocalization to neurophysiology. For example, it may not always be possible neurophysiologically to probe only horizontal flash position mislocalization or only horizontal saccades. This is because electrode positions in topographic maps may be in off-axis representations. More importantly, mislocalization has a two-dimensional landscape (Kaiser and Lappe, 2004). Thus, with our work, we provide an additional stepping stone toward direct neurophysiological experiments exploiting essentially identical behavioral tasks to those classically used in humans.

Indeed, we were motivated by the particularly strong backward mislocalization for upward saccades in humans (Grujic et al., 2018) and that we think deserves further investigation. We suspect that this phenomenon might be related to the asymmetry of representation in the superior colliculus between the upper and lower visual fields (Hafed and Chen, 2016; Zhang et al., 2022; Hafed, 2025), but this needs to be tested explicitly. In fact, besides recording activity related either to the saccade vector endpoint or to flash locations, we could make variants of our behavioral paradigm that can be more causal in nature. For example, we could replace the flash presentation with brief electrical microstimulation of the primary visual cortex or higher-level cortical areas. Similarly, we could replace the visually guided saccade with an electrically evoked saccade from the superior colliculus. This latter experiment would be particularly fascinating. For example, it is known that beyond a certain strength of electrical microstimulation in the superior colliculus, evoked saccades become more similar to each other under different stimulation parameters (Katnani and Gandhi, 2012). Thus, one could stimulate with two different “motor burst strengths” and ask how perisaccadic flashes are mislocalized under the two conditions (with the evoked saccades being the same). This would causally establish the functional role of sensory tuning in collicular motor bursts (Baumann et al., 2023) in perisaccadic perceptual phenomena that might, at least theoretically (Baumann et al., 2025), rely on saccade-related corollary discharge.

Similarly, we could perturb brain areas not implicated in corollary discharge and identify the limits of visual contributions to perisaccadic mislocalization. For example, if Monkey M's asymmetry in saccade peak speeds that we alluded to above was due to a region downstream of the superior colliculus, then it could be that the saccade motor commands (from the colliculus), and their associated (colliculus-related) corollary discharge, were the same for either rightward or leftward saccades in this monkey. If so, then the differences that we saw in the magnitude of perisaccadic mislocalization across hemifields in this animal would have been intrinsically visual in origin: the monkey would have had similar saccade commands (at the collicular level) but different speeds of retinal-image shifts.

The above example variants of our paradigm demonstrate why we think that this paradigm is useful for neurophysiology and also why we think that it can meaningfully add to a wealth of previous work exploring perisaccadic modulations in visual processing in the brain (Duhamel et al., 1992; Nakamura and Colby, 2002; Krekelberg et al., 2003; Kusunoki and Goldberg, 2003; Sommer and Wurtz, 2006; Churan et al., 2011, 2012; Zirnsak et al., 2014; Neupane et al., 2016a,b, 2020; Qian et al., 2022). These prior experiments did not exploit a simultaneous behavioral report by the animals, which our paradigm allows.

Of course, and as mentioned above, we do acknowledge that we did not explicitly test flash positions between the initial and final fixation positions associated with the saccade (which should show forward mislocalization). However, our use of multiple different saccade directions, coupled with the consistent backward mislocalization seen in all cases, makes us confident that what we saw was indeed “compression”-like mislocalization rather than the biphasic pattern present without visual references. Indeed, the vectors of mislocalization that we observed (Figs. 3, 5) as a function of time (relative to the saccade vector direction) were similar to those that we observed earlier in humans using multiple different saccade directions (Grujic et al., 2018). Thus, we believe that our results are consistent with those of Klingenhoefer and Krekelberg (2017). Importantly, our paradigm can be easily modified to also include flashes nearer to the initial fixation position than the saccade target.

We also agree with Klingenhoefer and Krekelberg (2017) that there can (and should) be quantitative differences in the parameters of the observed perisaccadic mislocalization between monkeys and humans. This is totally expected because factors like foveal magnification and sensory transduction speeds, and others, can be quantitatively different between the two species, even when they are qualitatively similar. Such quantitative differences are inevitable given the different brain sizes. What is important is that the qualitative equivalence is present, and this motivates using these animals in particular for invasive neurophysiological investigations.

One caveat with our work is that we relied on memory-guided saccades to report flash location. Such saccades have systematic errors in their landing positions (White et al., 1994; Willeke et al., 2019), which could also depend on the visual field location of the flashes (Hafed and Chen, 2016). Such saccades also have larger dispersions across trials (variable errors) than visually guided movements. Still, we chose to use memory-guided saccades because we did not want to impose any prior assumptions about where the monkeys were going to see the flashes. For example, if we had used “choice” targets, then we would have been imposing constraints on the spatial reports of the animals. This is problematic for a general two-dimensional paradigm like the one that we were trying to develop. Moreover, even manual pointing (via displayed cursors) by humans has systematic and variable errors (Willeke et al., 2019), and such pointing is generally used in mislocalization experiments (Grujic et al., 2018; Baumann et al., 2025). Thus, some amount of reporting errors is inevitable in any paradigm requiring a behavioral response from the subjects.

We also acknowledge that our choice to use memory-guided saccades could have come at the expense of having enough resolution to observe more subtle effects, like a potential dependence of perisaccadic mislocalization strength on the saccade target image appearance. Indeed, in parallel experiments with humans, we did find more systematic effects across most subjects than with the monkeys in the current study (Baumann et al., 2025). Thus, the inconsistency of the image dependence effects that we saw with our monkeys (Fig. 10) could reflect either added contamination by errors in memory-guided saccades, species differences from humans, or the need to test more monkeys with our current paradigm before reaching a conclusion about an image dependence of perisaccadic mislocalization in the animals.

One possibility to avoid the noise associated with memory-guided saccades could be to modify our paradigm slightly: we could adopt the approach of Klingenhoefer and Krekelberg (2017). That is, at the end of every trial, we would display a very dense two-dimensional array of grid positions, and the monkeys would simply choose which grid position matched the perceived flash location. We do not expect this to cause an increased training burden to the task. In any case, for the effects of saccade directions on mislocalization strengths, our paradigm was sufficient.

We also chose to not test presaccadic flashes. This was motivated purely by the pragmatic decision to increase data yield. By presenting flashes contingent on detecting a saccade, we were essentially ensuring obtaining a usable data point for every saccade trial accepted into the analysis. On the other hand, if we had randomized flash times independent of the saccadic reaction time, we would have needed to collect more trials and then recalculate flash times relative to saccade onset. This should work since it does not alter the paradigm dramatically, and it can also allow obtaining a full time course for perisaccadic mislocalization in monkeys. Such a time course can additionally distinguish between the compression and shift effects alluded to above, since the shift effects show a reversal in mislocalization direction between pre- and postsaccadic epochs (Honda, 1991, 1993). We have previously used both approaches (saccade-contingent or full time course) successfully in our experiments on perisaccadic visual suppression, in both humans and monkeys (Hafed and Krauzlis, 2010; Chen and Hafed, 2017; Idrees et al., 2020), and also in our perisaccadic perceptual mislocalization experiments in humans (Grujic et al., 2018; Baumann et al., 2025). Having said that, one has to carefully interpret trials in which the flash happened too long before saccade onset, especially because such a condition gives rise to a complete resetting of saccade plans (Buonocore and Hafed, 2023).

In all, we established a robust, two-dimensional perisaccadic mislocalization paradigm that is particularly useful for neurophysiological experiments with macaque monkeys.

## Synthesis

Reviewing Editor: David Schoppik, New York University Grossman School of Medicine Decisions are customarily a result of the Reviewing Editor and the peer reviewers coming together and discussing their recommendations until a consensus is reached. When revisions are invited, a fact-based synthesis statement explaining their decision and outlining what is needed to prepare a revision will be listed below. The following reviewer(s) agreed to reveal their identity: Suresh Krishna. Note: If this manuscript was transferred from JNeurosci and a decision was made to accept the manuscript without peer review, a brief statement to this effect will instead be what is listed below.

1. The idea of a dark room when using a computer monitor needs to be clarified - adding a statement about the light from the monitor will suffice. Early experiments of course used LEDs or low-persistence phosphor oscilloscopes for this reason, as did van Wetter and van Opstal. Are the results from this experiment to be compared to human results from the dark or with visual references?

2. Building on point #1, we feel that the manuscript needs considerable revision to place the findings in the context of previous results. Specifically, the biphasic perisaccadic mislocalization pattern reported by Honda (from experiments in the dark) in several papers (1991, 1993, 1999 - all in Vision Research) is not mentioned here in this paper. This biphasic pattern roughly consists of a forward mislocalization pre-saccadically and a backward mislocalization post-saccadically. Instead, the authors discuss a backward mislocalization but seemingly independent of flash timing, and dependent only on flash position - this is more akin to the compression pattern reported by Ross and colleagues, Lappe and colleagues, and others. This literature needs to be discussed more carefully and the authors' views clarified. Furthermore, previous studies have also shown that perisaccadic mislocalization changes with the flash time relative to the saccade. Most (but not all) studies find that the shift part of the mislocalization is in the direction of the saccade for flashes close to the saccade onset time but in the opposite direction for flashes close to the saccade offset time. The authors, however, presented flashes 30, 70, and 130 ms AFTER the saccade onset only. Figure 2, panel A, indicates that the flashes were either close to the saccade offset or after the saccade offset; there were no flashes close to the saccade onset.

We do not know whether the reported mislocalization against the saccade direction is a compression toward the target (as the authors suggest) or a backward shift or the sum of a compression and a backward shift or even the difference between a compression and a forward shift. Without such a basic understanding of the psychophysical results, it is difficult to apply the paradigm to physiological investigations of the underlying neural mechanisms, which we took to be the main goal of the manuscript. We suggest that although the results are interesting, the manuscript would be considerably stronger if the authors could determine what type of mislocalization (shift vs compression) was measured. Failing that, we feel that the manuscript should be restructured to focus on the psychophysical findings and discuss limitations of the experimental design rather than presenting the design as a better paradigm for future physiological studies.

3. The manuscript is not clear as to whether all trials were analyzed or only successful trials. What were the error-rates as a function of probe-timing and position? Was there any pattern to the errors, given that the very flashes occurred close to the saccade end and in a period where flashes could be missed ? Do the errors indicate anything insightful regarding the monkeys' strategy on the task ? What about errors that suggested non-performance of the task ?

4. How was the actual probe flash time calculated, given that an 85 Hz display was used? Was a photodiode used to verify the actual flash time? Also, given that the saccade duration is variable (as a function of saccade amplitude), it would be useful to comment on whether there was any pattern to the localization error and the flash detection rate, as a function of the flash onset-time relative to the saccade offset.

5. The paper starts with the words "two-dimensional", and the authors even say that "more importantly, mislocalization has a two-dimensional landscape (Kaiser and Lappe 2004) - line 946). But the paper does not seem to separately report the error along the direction of the saccade, and orthogonal to it. In particular, I could not tell from the paper whether there was any sign of "compression" with an orthogonal component (Kaiser and Lappe), in addition to "backward mislocalization" along the saccade direction.

6. The probe flash is stated to appear at 30, 70 and 130 ms (roughly) after primary saccade onset, and therefore roughly intrasaccadically (close to primary saccade offset) and about 30 and 100 ms after primary saccade offset. The primary saccade is likely to have been followed by a corrective saccade on some trials. Using the data here with corrective saccades on some trials (but not others), can the different localization patterns for the 3 flash times be related to their appearance when the eye is not at the final position prior to the start of the second saccade ? Is there any dependence of second saccade localization error, on the eye-position at the time of probe appearance ?

7. On a related note, is there any dependence of the localization error of the baseline trials on the actual delay period preceding it? This is relevant because the baseline trials are being used to control for memory-errors inherent with memory-guided saccades (e.g. lines 521-524), but the delay from second saccade target offset to second saccade execution can be quite short in the baseline trials. The variation in delay period allows one to look at the decay of memory over a relevant time-scale, and its potential influence on interpreting the error decrease for flashes presented at 0, 40 and 100 ms after primary saccade onset. In general, the probe localization error is likely to be a complex function of several factors, and the goal of this paper is to isolate the component that is related to peri-saccadic perceptual mislocalization, distinct from the errors due to memory-decay with time, for example.

8. In figures 4D, 3A etc, please plot the actual probe flash positions to easily visualize the backward mislocalization. It is not possible to spot any pattern in such plots with clouds of superimposed points - a few outliers dominate the visual percept and the numerous points on top of each other do not differentiate themselves. Adding a mean and confidence ellipse or error-bar will help make any point regarding mean and variability that the authors want to make.

9. The statement "Note that for each flash location, there was generally a smaller difference in baseline report locations between upward and downward saccades than the difference in mislocalization on strength (arrow length)." in Figure 4's legend is not convincing. We did not see that effect very clearly and additional quantification and explanation is necessary.

10. In the population analysis of this, the manuscript uses the metric - Euclidean distance to baseline report - to try and control for effects related to memory-guided errors; however, this metric does not control for various patterns of errors - for example alone, it leaves open the possibility that all of the localization errors with respect to flash location are identical (or even veridical), but it is the baseline report itself that is in error. While it is clear from the examples that this may not be the case, and having looked at all the data, the authors may be sure it is not the case, the analysis shown here leaves the possibility open, and is therefore incomplete. Some additional metrics (like Euclidean distance to actual probe location, perhaps) are additionally needed here as controls for the population data (even if only analyzed in the main text or in Supplementary material). We note that there might be interesting effects here given the points for 130 ms in monkey F in Figure 5A.

This point becomes more serious when looking at lines 760-770. The manuscript makes a point that the errors in baseline report (relative to true flash location) are of opposite sign to the errors in mislocalization amplitude (of second saccade endpoint relative to baseline report; we think this the measure since the manuscript refers to Figure 5B on line 762). However, we feel that this argues the opposite. If the probe localization error (relative to actual flash location) is identical in two conditions, but the baseline report error (Relative to flash location) is smaller in condition A, then the probe localization error relative to baseline report can easily come out to be longer in condition A (in many geometric arrangements of baseline report and second saccade endpoint, including the ones depicted in the examples). This issue needs to be dealt with carefully - the manuscript seems to be trying to control for a very restricted confound (lines 752-756), but the actual set of potential confounds here is larger.

These points are also true for Figure 7.

9. The manuscript should note that in Figure 6, the probes are in the upper visual field for upward saccades and in the lower visual field for downward saccades. Is there both a saccade direction effect and a visual field effect, or only the latter?

Minor

1. Missing reference information - Grujic N, Brehm N, Gloge C, Zhuo W, Hafed ZM (2018) Peri-saccadic perceptual mislocalization is different for upward saccades. J Neurophysiol.

2. It is unclear from Figure 1 how the delay period is timed - is it 500-800 ms from the acquisition of the saccade target by the eye, defined by entry into a target window ? Something else ?

3. (from the reviewing editor only) I would be remiss if I didn't point out that, as a graduate student, I did do a bit of work in this space that perhaps the authors might find worth considering: https://doi.org/10.1523/JNEUROSCI.1719-06.2006 I leave this entirely up to the authors

## Author Response

Responses to Reviewer Comments on: "Two-dimensional perisaccadic visual mislocalization in rhesus macaque monkeys" eN-TNWR-0547-24X March 5, 2025 We sincerely thank the editors and reviewers for their thoughtful and constructive feedback, which has greatly helped us improve the quality and clarity of our manuscript.

We have now fully addressed all of the reviewer comments. Briefly:

- We clarified the context of the study with respect to the literature, especially the early Honda studies in complete darkness - We exhaustively analyzed the systematic baseline errors, convincingly demonstrating that they were largely independent of saccade direction, unlike our mislocalization patterns; the newly added analyses strongly support our decision to use the baseline reports as our reference for veridical percepts - We further supported the "two-dimensional" aspect of our paradigm by adding analyses of orthogonal and parallel mislocalization - We included further details on potential effects of memory interval lengths, corrective saccades, eye positions at flash onsets, and the timings of visual mislocalization relative to saccade offset. We also analyzed the trial failure patterns of the monkeys. In all cases, we confirmed that our effects were genuine perisaccadic mislocalization phenomena that were not trivially explained by a great variety of potential other factors Synthesis Statement for Author (Required):

1. The idea of a dark room when using a computer monitor needs to be clarified - adding a statement about the light from the monitor will suffice. Early experiments of course used LEDs or low-persistence phosphor oscilloscopes for this reason, as did van Wetter and van Opstal. Are the results from this experiment to be compared to human results from the dark or with visual references? We sincerely apologize for this. To avoid confusion, we have removed the mention of a dark room in line 164. Our experiments were conducted using a brightly-illuminated monitor, which provided clear visual references. Therefore, our results should be compared to human data obtained under similar conditions with visual references (e.g. the work of Ross, Lappe, Krekelberg, and others). This is now explicitly mentioned in multiple places in the revised manuscript (e.g. lines 192-195, 218-219, 1020, 1030-1031).

2. Building on point #1, we feel that the manuscript needs considerable revision to place the findings in the context of previous results. Specifically, the biphasic perisaccadic mislocalization pattern reported by Honda (from experiments in the dark) in several papers (1991, 1993, 1999 - all in Vision Research) is not mentioned here in this paper. This biphasic pattern roughly consists of a forward mislocalization pre-saccadically and a backward mislocalization post-saccadically. Instead, the authors discuss a backward mislocalization but seemingly independent of flash timing, and dependent only on flash position - this is more akin to the compression pattern reported by Ross and colleagues, Lappe and colleagues, and others. This literature needs to be discussed more carefully and the authors' views clarified. Furthermore, previous studies have also shown that perisaccadic mislocalization changes with the flash time relative to the saccade. Most (but not all) studies find that the shift part of the mislocalization is in the direction of the saccade for flashes close to the saccade onset time but in the opposite direction for flashes close to the saccade offset time. The authors, however, presented flashes 30, 70, and 130 ms AFTER the saccade onset only. Figure 2, panel A, indicates that the flashes were either close to the saccade offset or after the saccade offset; there were no flashes close to the saccade onset.

We do not know whether the reported mislocalization against the saccade direction is a compression toward the target (as the authors suggest) or a backward shift or the sum of a compression and a backward shift or even the difference between a compression and a forward shift. Without such a basic understanding of the psychophysical results, it is difficult to apply the paradigm to physiological investigations of the underlying neural mechanisms, which we took to be the main goal of the manuscript. We suggest that although the results are interesting, the manuscript would be considerably stronger if the authors could determine what type of mislocalization (shift vs compression) was measured. Failing that, we feel that the manuscript should be restructured to focus on the psychophysical findings and discuss limitations of the experimental design rather than presenting the design as a better paradigm for future physiological studies.

We appreciate the reviewers' suggestion to better contextualize our findings within previous work on perisaccadic mislocalization, particularly the biphasic mislocalization pattern over time described by Honda. In response, we have now introduced significant revisions to the introduction (lines 98-109) to explicitly acknowledge that mislocalization in complete darkness follows a biphasic pattern, with a forward shift occurring when flashes are presented before or at saccade onset and a backwards shift occurring when flashes are presented after saccade completion. We had already hinted to this in the original introduction, including with one citation to Honda, 1989. However, we now see, and agree, that we should have done a much better job in this regard. This issue is now amended.

Additionally, we have added a new paragraph in the discussion to clarify the distinction between shifts and compressions in relation to our experiment (lines 1030-1050). Briefly, while previous studies in complete darkness have reported a biphasic mislocalization pattern with a reversal shortly after saccades, we are strongly convinced that our results reflect instead the compression-like effects of Ross, Lappe, Krekelberg, and others. For example, when we performed a more detailed time course analyses (as per the request of a later point below), our mislocalization strengths were always decreasing with increasing times (please see Extended Data Fig. 4-2). If we were observing a biphasic reversal of mislocalization, then mislocalization strength should have increased as we moved forward in time from saccade onset before decreasing again towards recovery (Honda 1991). More importantly, all studies that used bright displays (or even simpler visual reference frames) did not see a biphasic milocalization pattern. Rather, they saw compression-like patterns (e.g. Ross, Lappe, Krekelberg, Grujic, etc). This was also the case for Honda (1993) when using a (simple) visual reference frame around the saccade target.

By clarifying the above points, we aimed to provide a clearer link between our findings and the existing literature, while also reinforcing the rationale behind our paradigm design as a tool for investigating the neural mechanisms underlying perisaccadic mislocalization in structured visual environments (our primary interest). We appreciate the reviewers' insightful comments and believe that these revisions have improved the clarity and impact of our manuscript.

3. The manuscript is not clear as to whether all trials were analyzed or only successful trials. What were the error-rates as a function of probe-timing and position? Was there any pattern to the errors, given that the very flashes occurred close to the saccade end and in a period where flashes could be missed? Do the errors indicate anything insightful regarding the monkeys' strategy on the task? What about errors that suggested non-performance of the task? We have now clarified in the manuscript that only successful trials were analyzed (lines 291- 293, 613-14,647-648). Additionally, we have now conducted a detailed failure analysis for the unsuccessful trials. Briefly, we defined unsuccessful trials as those in which the monkey did successfully initiate the trial by making the first instructed saccade (and triggering the probe flash), but later failed in obtaining reward (e.g. by not successfully waiting for the go signal, etc). Other failures in which the monkey did not even initiate the trials at all were excluded from this analysis because they were not relevant for understanding the task performance per se; instead, they were more likely due to lack of motivation to fixate the initial fixation spot, or due to blinks or other eye position instabilities while attempting to fixate the initial fixatin spot. The results of our new failure analyses are now reported in lines 613-648.

To summarize, all three monkeys successfully completed more than 75% of the initiated trials, indicating that the probe flash was well visible across conditions. Failures fell into two main categories: (1) breaking fixation, either before the go signal for the report saccade or while maintaining fixation at the final location after the reporting saccade; and (2) failing to land within the predefined window for the report saccade. Both error types occurred at comparable rates across all animals. Moreover, the second type of error, failing to land within the predefined window, was more frequent in trials with shorter probe flash delays (again in all animals). This is particularly interesting as it aligns with the idea of observing the strongest mislocalization effects for these specific trials. In other words, had we made the tolerance windows even bigger, the monkeys would have likely been successful on those few trials. This observation also supports our interpretation that the monkeys' responses were influenced by the perceived, rather than the veridical, locations of the probes.

4. How was the actual probe flash time calculated, given that an 85 Hz display was used? Was a photodiode used to verify the actual flash time? Also, given that the saccade duration is variable (as a function of saccade amplitude), it would be useful to comment on whether there was any pattern to the localization error and the flash detection rate, as a function of the flash onset-time relative to the saccade offset.

We used the Psychophysics Toolbox for display control. This gave us exact time stamps at every frame update, ensuring accurate synchronization with the refresh cycle of the display. Prior tests with photodiodes (during the first few years of existence for the Hafed laboratory) confirmed the accurate timings of the Psychophysics Toolbox time stamps, which is expected given that the toolbox directly accesses the vertical blanking signal of the graphics card. Offline, we then recalculated the probe flash times relative to the actual saccade onset times, as we mentioned in Methods. This information is now better clarified in lines 169-174.

As suggested, we also examined the relationship between localization error and flash onset time relative to saccade offset (see lines 478-484). This analysis is now presented as Extended Data Fig. 4-2 and in lines 761-766. Specifically, we now binned our flash times more finely than we had originally done (i.e. within each of our three coarse flash times, we introduced more fine-grained temporal binning, which was afforded to us by variability in saccade durations). This revealed a continuous decreasing pattern of mislocalization strength with increasing time, as expected from compression-like effects (see above). Thus, aligning on saccade offset allowed us more fine-grainted time analyses, which were consistent with a gradual recovery from peak mislocalization.

Finally, concerning the term "flash detection rate", we are not fully sure what you mean here exactly. However, as mentioned in the response to the previous comment, the revised manuscript now reports failures of type (2), in which the monkey was not successful in placing gaze within the spatial tolerance window around the flash location. These failures were slightly more likely with the shortest flash times than with the later two flash times (but such errors were anyway a very small minority of all trials, as mentioned above).

5. The paper starts with the words "two-dimensional", and the authors even say that "more importantly, mislocalization has a two-dimensional landscape (Kaiser and Lappe 2004) - line 946). But the paper does not seem to separately report the error along the direction of the saccade, and orthogonal to it. In particular, I could not tell from the paper whether there was any sign of "compression" with an orthogonal component (Kaiser and Lappe), in addition to "backward mislocalization" along the saccade direction.

This is a good point. Please note that we had originally invoked the term "two-dimensional" in a slightly broader context, which also included the one in which we could replicate perisaccadic mislocalization for any arbitrary saccade direction and not just for horizontal saccades. However, we fully agree that our original analyses may have missed a great opportunity to indeed explore the two-dimensional landscape of mislocalization.

Therefore, in response, we conducted an additional analysis (Fig. 8 and lines 538-545 and 919-924) in which we separately examined the localization error along the saccade direction (panel B-E) and also along the component orthogonal to it (panel F-I). This is very similar to what we had done recently with human mislocalization patterns (Baumann et al., 2024). While the mislocalization effect was numerically larger along the saccade direction (as expected), the dependencies that we described in the manuscript held for both components individually. This analysis confirms that the reported effects were not limited to a single dimension, but were instead consistent across both the parallel and orthogonal components of mislocalization.

6. The probe flash is stated to appear at 30, 70 and 130 ms (roughly) after primary saccade onset, and therefore roughly intrasaccadically (close to primary saccade offset) and about 30 and 100 ms after primary saccade offset. The primary saccade is likely to have been followed by a corrective saccade on some trials. Using the data here with corrective saccades on some trials (but not others), can the different localization patterns for the 3 flash times be related to their appearance when the eye is not at the final position prior to the start of the second saccade? Is there any dependence of second saccade localization error, on the eye-position at the time of probe appearance? To address this comment, we explicitly examined the timings of corrective saccades, as well as the influences of eye position at the time of the probe flash (see lines 486-503).

First, we analyzed the temporal distribution of corrective saccades relative to the initial saccade onset. As shown in Extended Data Fig. 4-4, nearly all corrective saccades occurred after the probe flash presentations (as expected from saccadic refractory periods). Moreover, the timing of corrective saccades was very similar for flash times 1 and 2. For flash time 3, corrective saccades were slightly delayed. This delay is expected because of the phenomenon of saccadic inhibition, where the probe flash transiently suppresses the execution of corrective saccades in close temporal proximity (e.g. Khademi et al. 2023; Buonocore and Hafed, 2023). The presence of saccadic inhibition further supports the idea that the monkeys were indeed attending to the probe flash (e.g. White and Rolfs, 2016).

To further investigate whether eye position at the time of the probe flash influenced mislocalization, we also plotted the distance of the eye position from the saccade target at the moment of the probe flash against the mislocalization error (Extended Data Fig. 4-3). Negative values indicate instances where the eye was positioned between the initial fixation point and the saccade target; positive values indicate overshoots. We found that for the shortest flash times (30 ms), there were more negative than positive eye position values on the x-axis; this is expected because it indicates intrasaccadic flashes. For the later flash times, the eye positions were equally distributed around the true saccade target location, as expected (and also because we performed vector matching in our inclusion criteria). Most importantly, across all three monkeys, we did not observe any systematic relationship between eye position at flash time and mislocalization strength (even for the intrasaccadic flashes). For example, even though the distributions of eye positions for flash times 2 and 3 were very similar to each other (because the saccade had already ended for these two flash times), there was a large difference in mislocalization strength between these two conditions. Thus, probe flash time relative to the primary saccade exerted a much stronger influence on localization performance than the exact eye position at the moment of flash presentation. This is also true for human perisaccadic perceptual mislocalization effects.

These results are now described in several places in the revised manuscript (e.g. lines 768- 792).

7. On a related note, is there any dependence of the localization error of the baseline trials on the actual delay period preceding it? This is relevant because the baseline trials are being used to control for memory-errors inherent with memory-guided saccades (e.g. lines 521- 524), but the delay from second saccade target offset to second saccade execution can be quite short in the baseline trials. The variation in delay period allows one to look at the decay of memory over a relevant time-scale, and its potential influence on interpreting the error decrease for flashes presented at 0, 40 and 100 ms after primary saccade onset. In general, the probe localization error is likely to be a complex function of several factors, and the goal of this paper is to isolate the component that is related to peri-saccadic perceptual mislocalization, distinct from the errors due to memory-decay with time, for example.

We investigated whether localization error in baseline trials depended on the delay period duration preceding report saccade execution (see lines 466-476). As shown in Extended Data Fig. 4-1 and described in lines 744-759, there was a very subtle increase in localization error with longer delay durations. In this figure, we plotted the delay between target offset and the go signal against the Euclidean distance of the reported location from the actual target position on the screen (for baseline trials). While there was a very slight trend of increasing error with longer delays, the effect was minimal and did not appear to be a major confound in our interpretation of perisaccadic mislocalization effects. In fact, in the same figure, we added the mislocalization reports on perisaccadic flash trials, which had much larger error magnitudes than the baseline errors (even if baseline errors were extrapolated much longer in time).

Indeed, prior studies on memory-guided saccades have also demonstrated very minor (if at all) memory decays in the systematic errors that were observed. For example, White et al. (1994, https://doi.org/10.1016/0042-6989(94)90259-3) documented that systematic errors in memory-guided saccades remained relatively stable for delays of up to 5600 ms. We also found this in human subjects as well (e.g. Willeke et al., 2022), and we think that this has to do more with how the SC network might behave during working memory (e.g. Hafed, 2025). All of these findings suggest that while memory decay might introduce a very minor contribution to localization errors over extended delays, it is highly unlikely to account for the error dynamics observed in our perisaccadic mislocalization conditions. By including baseline trials in our analyses, we ensured that the mislocalization effects reported in our study were primarily driven by perisaccadic perceptual distortions rather than by general memory-related systematic errors (also see the new Fig. 4 and below for further evidence supporting our view).

8. In figures 4D, 3A etc, please plot the actual probe flash positions to easily visualize the backward mislocalization. It is not possible to spot any pattern in such plots with clouds of superimposed points - a few outliers dominate the visual percept and the numerous points on top of each other do not differentiate themselves. Adding a mean and confidence ellipse or error-bar will help make any point regarding mean and variability that the authors want to make.

We apologize for this. The actual probe flash positions were indeed indicated, but in gray. This was a poor choice. Therefore, we have now updated all figures such that the true flash locations were indicated with bright pink squares. Please also note that for each raw plot, we had a neighboring panel showing mean and error bar indications, to better illustrate the backward mislocalization patterns, as well as the variability of the data. However, now, we also added bright cyan crosses in any panel with raw plots, indicating the mean of each cluster. These adjustments hopefully enhance clarity and make it easier to interpret the underlying trends in the data.

9. The statement "Note that for each flash location, there was generally a smaller difference in baseline report locations between upward and downward saccades than the difference in mislocalization on strength (arrow length)." In Figure 4's legend is not convincing. We did not see that effect very clearly and additional quantification and explanation is necessary.

We apologize for this. The original intent was that this idea was to be explicitly covered (later) by the actual quantifications in the original Fig. 6. However, we now realize that this was a poor choice since the original Fig. 6 came much later in the text. Thus, we have now added a new panel to the figure, directly checking the baseline report differences (see the new Fig. 5D and lines 833-838). Specifically, we plotted the difference in Euclidean distance between the reported and actual target locations for baseline trials as bars, and we also compared it to the difference observed for probe flash time 1. The results clearly demonstrated that the difference in baseline reports between upward and downward saccades was considerably smaller than the difference observed in the probe flash condition. This supports the interpretation that the interaction between saccade direction and probe flash timing drives the observed mislocalization effects, rather than an overall bias in reporting after upward versus downward saccades. Please also see the new Fig. 4, which convincingly justifies our choice to use baseline reports as the reference for veridical percepts.

10. In the population analysis of this, the manuscript uses the metric - Euclidean distance to baseline report - to try and control for effects related to memory-guided errors; however, this metric does not control for various patterns of errors - for example alone, it leaves open the possibility that all of the localization errors with respect to flash location are identical (or even veridical), but it is the baseline report itself that is in error. While it is clear from the examples that this may not be the case, and having looked at all the data, the authors may be sure it is not the case, the analysis shown here leaves the possibility open, and is therefore incomplete. Some additional metrics (like Euclidean distance to actual probe location, perhaps) are additionally needed here as controls for the population data (even if only analyzed in the main text or in Supplementary material). We note that there might be interesting effects here given the points for 130 ms in monkey F in Figure 5A.

We are very certain that we were indeed measuring perisaccadic mislocalization effects, and not artifacts of our use of the baseline reports as the reference for veridical reports. To further demonstrate this, we have now conducted an additional analysis (see lines 448-459) explicitly characterizing the systematic error patterns in the baseline trials, and how they were decidedly different from the peri-saccadic mislocalization errors (even when the latter were measured relative to true probe location, as suggested by the reviewers).

The results are now presented in Fig. 4 and lines 711-734. Briefly, for each primary saccade direction, we plotted the baseline report error vector; in the same plot, and for the same flash locations, we also illustrated the direction and amplitude of the mislocalization error vector for the first flash time (i.e. the condition with the biggest mislocalization error amplitude). For each monkey, the error in baseline saccades was highly systematic regardless of primary saccade direction. For example, in panel A of this new figure, baseline report saccades were always biased upward (and were of approximately the same size), independent of primary saccade direction. On the other hand, the mislocalization vector was always opposite the primary saccade vector direction. We also explicitly quantified this large and qualitative difference between baseline and mislocalization errors: specifically, we measured the angle of the baseline or mislocalization report vector relative to the angle of the primary saccade direction. Because the baseline error vectors were systematic and independent of primary saccade direction, this measure yielded polar histograms that spanned the full 360 degress across primary saccade directions; instead, the mislocalization vectors were always clustered at 180 deg relative to primary saccade direction. Thus, our results were not explained by our decision to include baseline reports as the reference for veridical percepts.

In the same panel A of the same figure, we also convincingly illustrated why we were justified in using the baseline reports as our reference for the veridical percepts. Specifically, for some example primary saccade directions, we additionally indicated the direction of the mislocalization vector if it was measured relative to the baseline report endpoint rather than to the actual probe location (dashed arrows). Clearly, the 180-deg alignment with the primary saccade vector direction got even better. This makes perfect sense: if memory- guided saccades have a systematic error, then there is absolutely no reason for the report saccades in our mislocalization trials to also not suffer from the same systematic error. Please note that we did not show dashed arrows in panel A of the figure for all primary saccade directions, but this was only done to avoid cluttering the figure. Otherwise, visual inspection of the figure clearly shows that even the directions not shown with dashed arrows behaved like we demonstrated for the other directions.

In all, the systematic difference in angular error in baseline and report trials indicates that the mislocalization effect cannot simply be attributed to a pre-existing bias in baseline reports. Instead, our paradigm suggests that the animals were genuinely reporting the perceived location of the probe flash. Consequently, any baseline bias must also manifest in the mislocalized reports, and it was therefore crucial to account for this baseline bias when quantifying mislocalization.

This point becomes more serious when looking at lines 760-770. The manuscript makes a point that the errors in baseline report (relative to true flash location) are of opposite sign to the errors in mislocalization amplitude (of second saccade endpoint relative to baseline report; we think this the measure since the manuscript refers to Figure 5B on line 762). However, we feel that this argues the opposite. If the probe localization error (relative to actual flash location) is identical in two conditions, but the baseline report error (Relative to flash location) is smaller in condition A, then the probe localization error relative to baseline report can easily come out to be longer in condition A (in many geometric arrangements of baseline report and second saccade endpoint, including the ones depicted in the examples). This issue needs to be dealt with carefully - the manuscript seems to be trying to control for a very restricted confound (lines 752-756), but the actual set of potential confounds here is larger.

These points are also true for Figure 7.

Please note that we were referring to the *difference* in baseline reports between upward and downward saccades as having a different sign from the *difference* in mislocalization reports. Thus, we were isolating the effect of the mislocalization's dependence on saccade direction, and saying that any differential in it (as a function of saccade direction) was not existing also as a differential in the baseline reports. This was very important because it helped demonstrate that the differential was specific to the mislocalization itself.

Please also note that we observed clear mislocalization even when viewed completely independently of any baseline reports. For example, all of our raw plots showed clear mislocalization even if we had not plotted the baseline reports at all (e.g. Figs. 3, 5). Similarly, the new figure added in response to the previous comment above (Fig. 4) convinces that baseline reports were indeed a very suitable reference frame for measuring the veridical percepts, because they allowed discounting the influences of systematic errors in memory-guided saccades.

Finally, we explicitly addressed this current point even further with yet one more analysis. Specifically, we now measured all mislocalization reports relative to the reports on flash time 3, instead of relative to baseline reports. Since there is clear perceptual recovery in perisaccadic mislocalization, we could now use flash time 3 as the reference frame being closest to the actual final recovery in the percept. When we did this, we found that there was still clear mislocalization at flash times 1 and 2 (as expected from all of our other analyses including the raw plots), and that there was still stronger mislocalization for upward saccades. Thus, our results were robust even when not using baseline reports at all as our reference frames (please see lines 531-536 and 885-896).

9. The manuscript should note that in Figure 6, the probes are in the upper visual field for upward saccades and in the lower visual field for downward saccades. Is there both a saccade direction effect and a visual field effect, or only the latter? We agree. We now clarify in line 553-560 that in our paradigm, an upward saccade was also always associated with a probe flash location in the upper visual field, and vice versa for downward saccades. For oblique saccades, some flash locations were at the same vertical eccentricity as the saccade target, but they were still in the upper visual field presaccadically when the oblique saccade was upward, and still in the lower visual field presaccadically when the oblique saccade was downward. This is also an issue that affected human experiments exploring differences in mislocalization as a function of saccade direction (Grujic et al., 2018). For example, in Grujic et al., 2018, there was a clear interaction for oblique saccades between flash locations and saccade directions.

Minor 1. Missing reference information - Grujic N, Brehm N, Gloge C, Zhuo W, Hafed ZM (2018) Peri-saccadic perceptual mislocalization is different for upward saccades. J Neurophysiol.

We have now added the missing reference information in line 1231 2. It is unclear from Figure 1 how the delay period is timed - is it 500-800 ms from the acquisition of the saccade target by the eye, defined by entry into a target window? Something else? We have now clarified in line 223-224 that the delay period started when the monkeys' gaze entered the saccade target window.

3. (from the reviewing editor only) I would be remiss if I didn't point out that, as a graduate student, I did do a bit of work in this space that perhaps the authors might find worth considering: https://doi.org/10.1523/JNEUROSCI.1719-06.2006 I leave this entirely up to the authors In our revised markup, we have now added more text about the importance of saccade goals in reference frame updating, and this has allowed us to also cite this interesting study (please see lines 105-109).
