# Supplementary figures and images for: Two-Dimensional Perisaccadic Visual Mislocalization in Rhesus Macaque Monkeys
Source: eNeuro. 2025 Jun 4;12(6):ENEURO.0547-24.2025. doi: 10.1523/ENEURO.0547-24.2025 (PMC12143751; doi:10.1523/ENEURO.0547-24.2025)

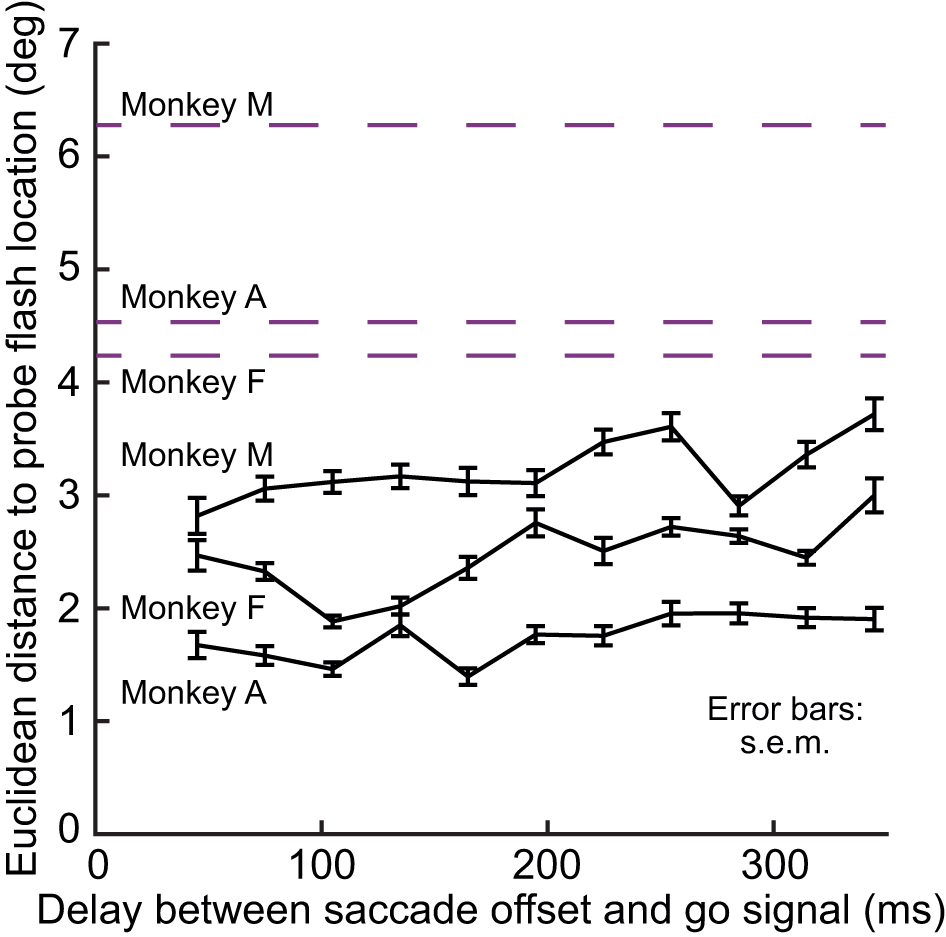

Supplement: Figure 4-1 — Relationship between delay period duration and baseline localization error. Euclidean distance between the reported location and the actual probe flash position in baseline trials as a function of delay duration between primary saccade offset and the go signal for the report saccade. Each data point represents mean localization error per time bin, with error bars indicating SEM. The dashed purple lines indicate the peak mislocalization values for the individual monkeys, providing a reference for the maximal observed perceptual distortions in the perisaccadic period. Even with extrapolation for much longer delays, the baseline report errors were much smaller than actual perisaccadic mislocalization strengths. Download Figure 4-1, TIF file. [file eneuro-12-ENEURO.0547-24.2025-s001.tif]

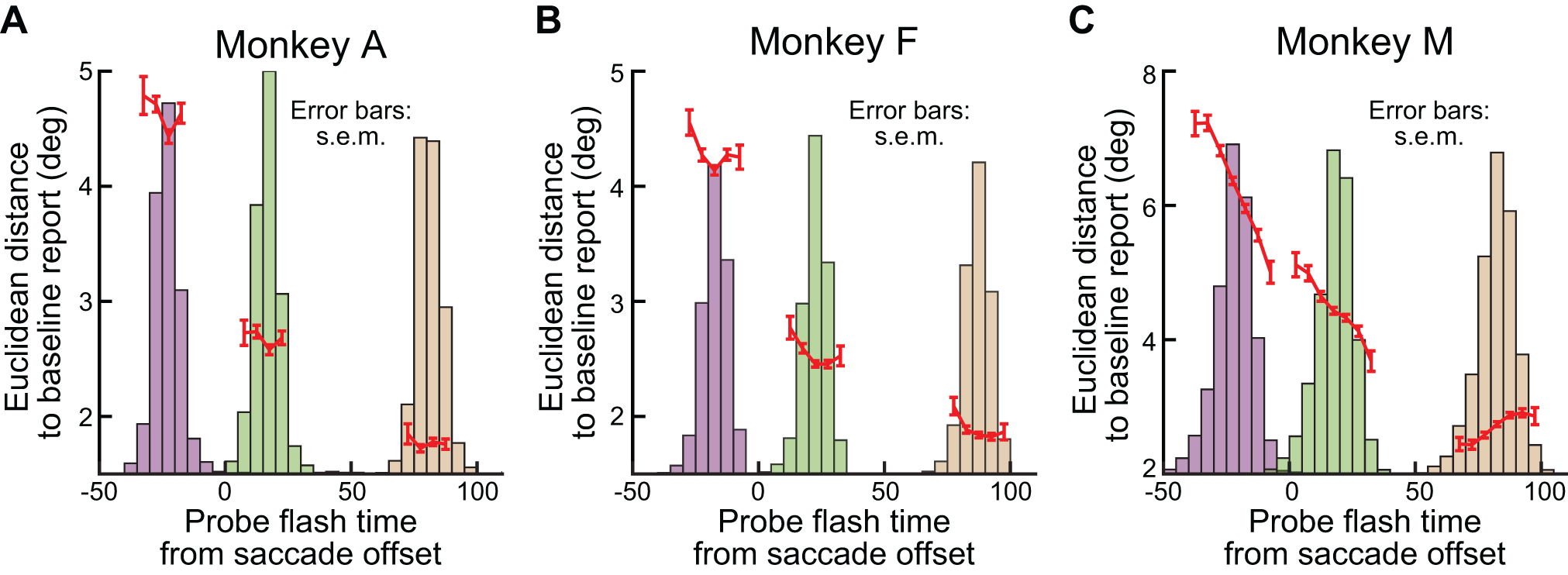

Supplement: Figure 4-2 — Time course of perisaccadic mislocalization relative to saccade offset. (A-C) Euclidean distance to the baseline report as a function of probe flash onset time relative to saccade offset for each monkey (red). Mislocalization gradually decreased with increasing delay after saccade offset, indicating a recovery of veridical localization over time. Error bars represent SEM. The faint histograms in the background show the actual underlying probe flash times; note that we did not plot red data points if there were fewer than 100 observations in any histogram bin. Download Figure 4-2, TIF file. [file eneuro-12-ENEURO.0547-24.2025-s002.tif]

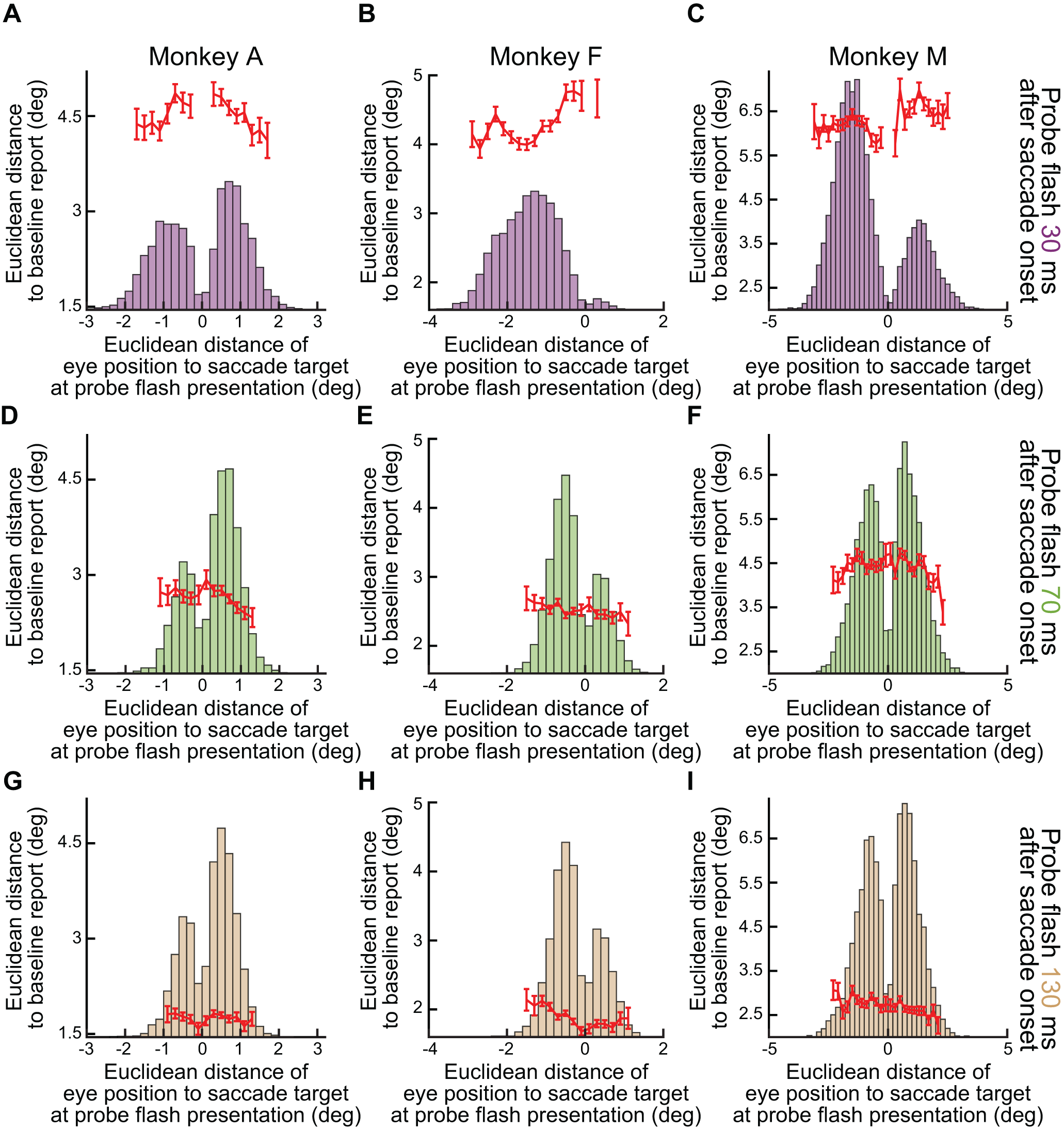

Supplement: Figure 4-3 — Eye position at the time of probe flash presentation and its relationship to mislocalization strength. (A-C) Euclidean distance (red) to the baseline report as a function of eye position at the time of probe flash presentation for probe flashes occurring 30 ms after saccade onset for the individual monkeys. (D-F) The same relationship for probe flashes occurring 70 ms after saccade onset. (G-I) The same analysis for probe flashes occurring 130 ms after saccade onset. In each case, the faint histograms in the background show the bins in which we had eye position data to document. Across all three monkeys and all time points, no systematic relationship was found between eye position and mislocalization strength, confirming that localization errors were primarily driven by the timing of the flash rather than by momentary gaze position. Indeed, for each monkey, the mislocalization strength decreased much more strongly from the top to bottom row than within each row as a function of eye position. Download Figure 4-3, TIF file. [file eneuro-12-ENEURO.0547-24.2025-s003.tif]

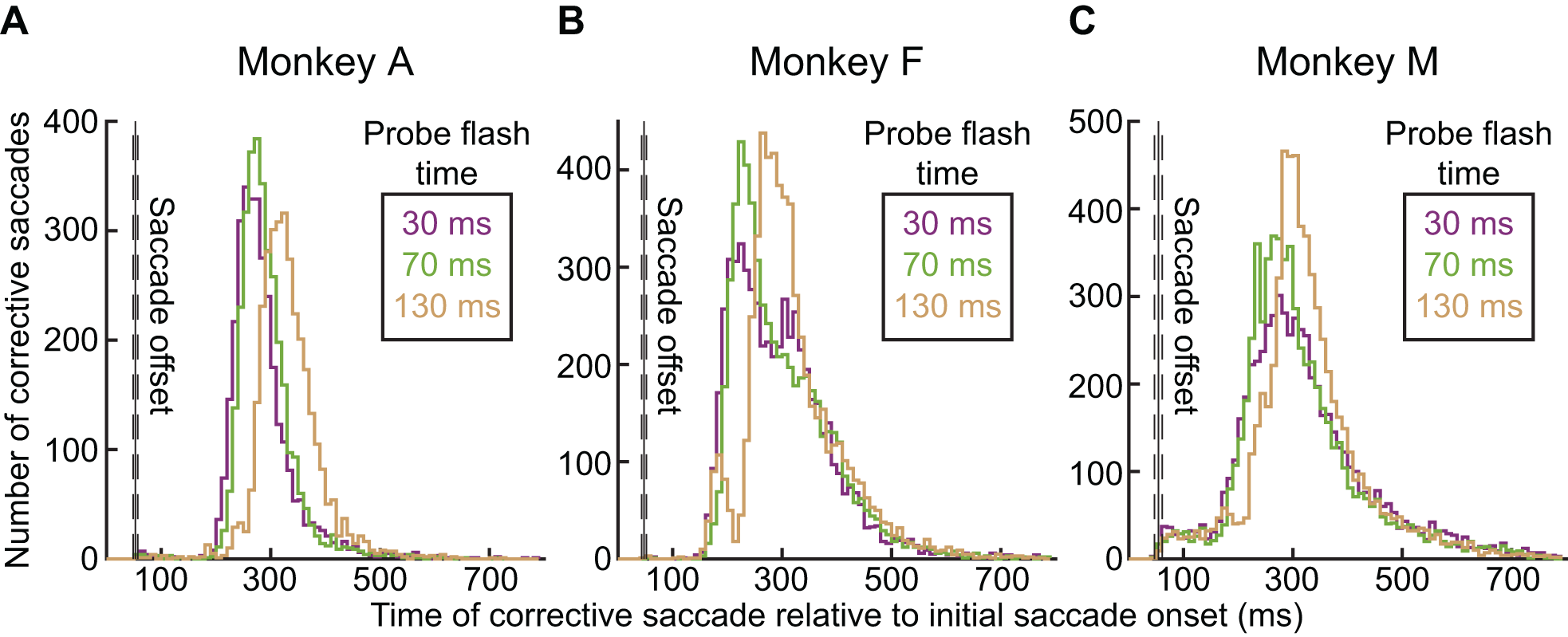

Supplement: Figure 4-4 — Corrective saccades were too late to account for mislocalization effects. (A-C) The temporal distribution of corrective saccades relative to the initial saccade onset for each monkey. The number of corrective saccades is plotted over time, aligned to the initial saccade onset. The different probe flash timings (30 ms, 70 ms, and 130 ms after saccade onset) are color-coded. The majority of corrective saccades occurred after probe flash presentation, confirming that they did not influence perisaccadic mislocalization. A slight delay in corrective saccades for late probe flash presentations (130 ms) suggests transient saccadic inhibition, supporting the notion that the monkeys attended to the probe flash. The vertical black lines with surrounding dashed lines indicate the mean and SEM times of saccade offset. Download Figure 4-4, TIF file. [file eneuro-12-ENEURO.0547-24.2025-s004.tif]
